# Phloretin decreases microglia-mediated synaptic engulfment to prevent chronic mild stress-induced depression-like behaviors in the mPFC

**DOI:** 10.7150/thno.76553

**Published:** 2023-01-16

**Authors:** Chenchen Li, Bo Liu, Jingyi Xu, Bin Jing, Lijie Guo, Liyong Wang, Milin Wang, Haochen Zhang, Qianqian He, Xin Yu, Yan Zhang, Zhi-Qing David Xu, Yutao Yang

**Affiliations:** 1 Department of Neurobiology, School of Basic Medical Sciences, Capital Medical University, Beijing, 100069, China.; 2 Department of Basic Medical Sciences, School of Medicine, Tsinghua University, Beijing 100084, China.; 3 School of Biomedical Engineering, Capital Medical University, Beijing,100069, China.; 4 Central lab, Capital Medical University, Beijing, 100069, China.; 5 Sports & Medicine Integration Research Center, Capital University of Physical Education and Sports, Beijing, 100086, China.; 6 Department of Pathology, School of Basic Medical Sciences, Capital Medical University, Beijing, 100069, China.

**Keywords:** Phloretin, stress, depression, microglia, synaptic engulfment

## Abstract

**Background:** Stress is an important risk factor to induce psychiatric disorders such as depression. Phloretin (PHL), a natural dihydrochalcone compound, has been shown to exhibit anti-inflammatory and anti-oxidative effects. However, the impact of PHL on the depression and the underlying mechanism remain unclear.

**Methods:** The animal behavior tests were used to determine the protective of PHL on the chronic mild stress (CMS)-induced depression-like behaviors. The Magnetic Resonance Imaging (MRI), electron microscopy analysis, fiber photometry, electrophysiology, and Structure Illumination Microscopy (SIM) were used to investigate the protective of PHL on the structural and functional impairments induced by CMS exposure in the mPFC. The RNA sequencing, western blot, reporter gene assay, and chromatin immunoprecipitation were adopted to investigate the mechanisms.

**Results:** We showed that PHL efficiently prevented the CMS-induced depressive-like behaviors. Moreover, PHL not only attenuated the decrease of synapse losses but also improved the dendritic spine density and neuronal activity in the mPFC after CMS exposure. Furthermore, PHL remarkably inhibited the CMS-induced microglial activation and phagocytic activity in the mPFC. In addition, we demonstrated that PHL decreased the CMS-induced synapse losses by inhibiting the deposition of complement C3 deposition onto synapses and subsequent microglia-mediated synaptic engulfment. Finally, we revealed that PHL inhibited the NF-κB-C3 axis to display neuroprotective effects.

**Conclusions:** Our results indicate that PHL represses the NF-κB-C3 axis and subsequent microglia-mediated synaptic engulfment to protect against CMS-induced depression in the mPFC.

## Introduction

Depression is the most common mental disorder which imposes great economic burdens on individuals, families and society 1. Increasing evidences have shown that chronic stress leads to the development of mental illnesses including depression 2-4. Although chronic antidepressant treatment, enriched environment as well as psychotherapy interventions have been shown to protect against the development of psychiatric disorders including depression by increasing the stress resilience 5-9, above interventions still have some limitations such as side effect and inconvenient execution, which in turn does not fully meet the requirement of stress vulnerable populations. Therefore, it is necessary to develop a safe, efficient and convenient compound to decrease the development of depression.

It is well documented that stress contributes to the development of psychiatric disorders by influencing neuronal plasticity 2, 10. Neurons in the prefrontal cortex and hippocampus are highly sensitive to stress and can be remodeled after stressor exposure 11. In the hippocampus, chronic stress not only leads to the shrinkage of dendrites of CA3 but also results in the loss of spines in CA1 neurons 12. In the rodent prefrontal cortex, chronic stress changes the dendritic morphology of pyramidal neurons and decreases the dendritic spine density and synaptic plasticity 13-15. In addition, a recent study has reported that social defeat stress selectively reduces the neuronal activity in the ventromedial prefrontal cortex 16.

Synapse loss is an early and important characteristic underlying brain circuit dysfunction in depression and neurodegenerative diseases 13. Increasing evidences have indicated that microglia and synapse interactions lead to synapse loss and dysfunction 17. Microglia are innate immune cells in central nervous system (CNS) that have been shown to respond to changes in brain homeostasis 18. In addition to release the cytokines and chemokines, microglia have been shown to mediate synapse engulfment through complement cascade in development and neurodegenerative diseases 17. Therefore, inhibition of excessive synapse losses mediated by complement cascade might be a potential approach to prevent the onset of stress-induced depression.

Phloretin (PHL) is a natural dihydrochalcone mainly isolated from apples and generally recognized as safe (GRAS) by the Flavor and Extract Manufactures' Association of the United States (FEMA). As a dietary flavonoid, PHL has various biological activities, including anti-cancer activity, anti-inflammatory effect, anti-oxidative activity, as well as prevention of cardiovascular disease 19. In addition to the above biological activities, PHL has been shown to exhibit protective effects in scopolamine-induced amnesia in mice 20 and cerebral ischemia/reperfusion in rats 21. However, whether PHL displays a potential anti-depression property in the CMS-induced depression-like behaviors has not been determined.

In this study, we first determined whether PHL could protect against stress-induced depression-like behaviors. Second, we examined whether PHL could improve the neural plasticity in the mPFC after exposure to CMS. Third, we investigated the protective role of PHL in CMS-induced microglial activation and phagocytic activity. Finally, we explored the mechanism underlying the protective effects of PHL on the microglia-mediated synaptic engulfment induced by CMS exposure in the mPFC.

## Materials and methods

### Animals

Male Sprague-Dawley (SD) rats (200 to 250 g) were housed in cages at 20 - 25 °C on a 12-h light/dark cycle with *ad libitum* access to food and water. Animals were habituated in the housing facilities in the first week prior to experiments. All experimental protocols were approved by the Animal Care and Use Committee of Capital Medical University (Permit Number: AEEI-2020-136) and were in accordance with National Institutes of Health Guide for the Care and Use of Laboratory Animals. According to the 3R (reduction, refinement, and replacement) principle of animal experimental strategies, we used the minimum number of animals to meet the statistical analysis requirements.

### CMS procedure

The CMS procedure used in this study was adopted from a previous study with slight modifications 22. Briefly, rats were exposed to a variety of randomized stressors over 3-week, including water/food deprivation, cold water swimming, restraint, reversed light-dark cycle, clip tail, and cage tilt. No same stressor was adopted in succession. Vehicle rats were remained undisturbed in their home cages under normal conditions except for necessary procedures, including routine cleaning and handling.

### Behavioral Tests

After stress or PHL treatment, rats were subjected to behavioral tests, including the sucrose preference test (SPT), the forced swimming test (FST), and the open field test (OFT). All above three behavioral tests were carried out according to our previous study 23. To avoid the influence of different tests, only one behavioral test was performed per day. The detailed procedures of the SPT, FST, and OFT were listed in supplementary materials.

### Detection of the brain distribution of PHL

Rats were intragastrical administered with PHL (50 mg/kg) and sacrificed after 15, 30, 60, or 120 min, respectively. Brain tissue samples were weighed and homogenized in 0.9% NaCl. Then, samples were centrifuged and supernatant was collected. An aliquot of 100 µL of brain sample was transferred to new centrifuge tube, and internal standard Reveratrol was added. Then, samples were centrifuged and the supernatant was injected into LC-MS/MS system (Shimadzu, Kyoto, Japan) and tandem AB SCIEX 6500 QTRAP mass spectrometer (Applied Biosystems, On, Canada) for analysis. The detailed procedures were provided in supplementary materials.

### MRI image acquisition and analysis

Before scanning, every rat was put into an inhalation chamber to have a preoperative anesthesia with 5% isoflurane. Then, the rat was positioned on the MRI scanner bed by a tooth bar. Next, the rat brain was scanned by a 7.0T MRI scanner (Bruker, Ettlingen, Germany). High resolution structural images were acquired using a RARE T2-weighted sequence. The mPFC volume was calculated with an atlas-based VBM-DARTEL method as described in our previous study 24. The detailed procedures were provided in supplementary materials.

### Electron microscopy analysis

The mPFC tissues were dissected out and put into 2.5% glutaraldehyde for 4 h at 4 °C. Then, tissues were post-fixed with 1% osmium tetroxide for 1 h. After dehydration with cold graded ethanol, the tissues were infiltrated by using a mixture of one-half propylene oxide overnight and then embedded in resin. Next, above tissues were sliced into ultrathin sections (70 nm thick) and stained with uranyl acetate and lead citrate. Images were captured with an electron microscope HT7700 (Hitachi, Tokyo, Japan) at 80 kV.

### Immunofluorescence and imagine analysis

Coronal sections of frozen rat brain sections were blocked with 10% fetal bovine serum (FBS) (Life Technologies, Carlsbad, CA, USA) in PBST (0.1 M PBS with 0.3% Triton X) at room temperature for 2 h. Brain sections were then incubated with primary antibodies in PBST with 10% FBS overnight at 4 °C. After washing 3 times with PBST for 5 min, sections were incubated with secondary antibodies for 2 h at 25 °C. Finally, all sections were washed with PBST for 3 times and mounted on slides with Fluoromount-G anti-fade medium containing DAPI (Thermo Fisher Scientific, Madison, WI, USA). The confocal and Structure Illumination Microscopy (SIM) techniques were used in subsequent image taken. The detailed procedures for imagine analysis were listed in supplementary materials.

### Fiber Photometry

A fiber photometry system (RWD, Shenzhen, Guangdong, China) was applied for recording Ca^2+^ signals from mPFC neurons. After the injection of 200 nl AAV-CamKIIα-GCaMP6s virus (BrainVTA, Wuhan, Hubei, China), an optical fiber was positioned immediately in a ceramic ferrule and inserted toward the mPFC (AP: 3 mm; ML: ± 0.5 mm; DV: -4 mm) through the craniotomy. The ceramic ferrule was supported with 3 ceramic screws and dental acrylic. After a period of 2-week of PHL pre-treatment followed 3-week of CMS exposure, the 1% sucrose water intake induced-changes of Ca^2+^ signals were recorded. Then, the fluorescence signals were collected and converted to Δ*F*/*F* (%) values. The detailed procedures were provided in supplementary materials.

### Electrophysiological experiments

Rats were anesthetized and perfused with cold artificial cerebrospinal fluid (ACSF). Brains were removed quickly and cut with the vibratome VT1200S (Leica, Wetzlar, Germany) in cold artificial cerebrospinal fluid. The brain slices were incubated in ACSF at 37 °C for 45 min and then recovered for 30 min at room temperature in ACSF. After recovery, slices were put into the recordings chamber and continuously perfused with oxygenated-standard ACSF. The membrane potential was held at -60 mV, and the mini excitatory postsynaptic currents (mEPSC) of layer 5 pyramidal neurons in mPFC were recorded. For the LTP and LTD recordings, brain slice was fixed on a MED64 probe (Panasonic, Osaka, Japan) and continually perfused with oxygenated-standard ACSF. After baseline responses stabilized for 10 min, LTP was induced with theta-burst stimulation, whereas LTD was induced with low frequency stimulation (LFS). LTP and LTD were recorded for 60 min, respectively. The detailed procedures were provided in supplementary materials.

### RNA sequencing

Total RNA was prepared from mPFC tissue using TRIzol Reagent (Invitrogen, Carlsbad, CA, USA) according to the manufacturer's protocols. The RNA-seq transcriptome library was prepared following the NEBNext® Ultra™ RNA Library Prep Kit for Illumina® (New England Biolabs, Ipswich, MA, USA) with 1 µg of total RNA. RNA sequencing was performed using a NovaSeq6000 and High Output v2 kit (Illumina, San Diego, CA, USA). All RNA-seq raw data were uploaded in the Gene Expression Omnibus database under accession number (GSE193284). The detailed procedures were listed in supplementary materials.

### Cell culture

BV2 murine microglial cells were maintained in Dulbecco's modified Eagle's medium (DMEM)/F12 (Corning, Manassas, VA, USA) supplemented with 10% heat-inactivated fetal bovine serum (FBS) (Life Technologies) and 1% penicillin-streptomycin solution (Life Technologies). The cells were maintained in a humidified incubator with 95% air in 5% CO_2_ at 37 °C. Rat primary microglia and astrocytes were prepared from 1-day-old SD rats. The detailed procedures were listed in supplementary materials.

### Plasmid construction

The 526-bp upstream regulatory sequence of the rat C3 gene was obtained by PCR from PC12 cell genomic DNA. The PCR products were purified with a gel extraction kit (Promega, Madison, WI, USA) and digested with* Kpn* I and *Hin*d III (NEB). Then, the digested products were inserted into pGL3-basic vector (Promega) by T4 DNA ligase (NEB). The resultant reporter plasmid was named as C3-Luc. The four mutant reporter plasmids, Mut1-Luc to Mut4-Luc, were constructed by recombinant PCR using C3-Luc as a template. The primer sequences were listed in supplementary materials.

### Reporter gene assay

BV2 cells were seeded in 24-well plates and grown to 70% confluency. Then, 100 ng of reporter plasmid and 20 ng of pRL-TK plasmid were co-transfected into BV2 cells by using Lipofectamine 3000 (Life Technologies). After cell transfection, cells were pre-treated with or without 20 µM of PHL for 24h, and then treated with 50 ng/ml of IL-1β for another 24h. Then, cells were lysed with passive lysis buffer provided in the Dual-Luciferase® Reporter Assay System (Promega). Finally, the Firefly and Renilla luciferase activities were examined by using the kit according to the instructions. The values are the mean ± SE and are normalized to Renilla luciferase activity.

### Chromatin immunoprecipitation (ChIP) assay

ChIP experiments were carried out according to the protocols provided with EZ-Magna ChIP™ A/G Chromatin Immunoprecipitation Kit (Millipore, Billerica, MA, USA). Briefly, 1×10^7^ cells were cross-linked with 1% formaldehyde, and the cross-linking reaction was stopped by glycine. Then, cells were lysed and sonicated into DNA fragments of 200 to 1,000 bp. Then, immunoprecipitation was performed using anti-p65 (CST, Boston, MA, USA) or control IgG serum (CST) with cell lysates. Then, real-time PCR was performed with DNA extracted from the immunoprecipitated chromatin. The detailed procedures and the primers for the p65 binding site in the rat C3 promoter were listed in supplementary materials.

### Stereotaxic surgery and cannula placement

Rats were anesthetized and head-fixed in a stereotactic apparatus (RWD). For cannula implantation, the rat skull was exposed and a small hole was drilled using a dental drill around the region of interest. Then, the stainless steel guide cannulas (RWD) were bilaterally implanted into the mPFC region. Stainless steel obturators were inserted into the guide cannulas until an injection was to be performed. When the intracranial injection was started, the obturators were removed and injection cannulas were inserted. After injection, the cannulas were left for another 5 min to allow drug diffusion. The detailed procedures were listed in supplementary materials.

### Statistical Analysis

Data were presented as mean ± SEM and analyzed with GraphPad Prism (NIH). The normality of the data distributions was analyzed by D'Agostino-Pearson omnibus test, and the data lies outside two standard deviations were excluded. Data that obey normal distribution were used for subsequent statistical analysis. Statistical significance for group comparisons was examined by one-way or two-way ANOVA followed by the Turkey's method for post hoc multiple comparison tests. Two-tailed t tests were adopted to compare the differences between two groups. Differences with *P* < 0.05 were considered statistically significant.

## Results

### PHL attenuates the CMS-induced depressive-like behaviors in rats

**Figure 1:**
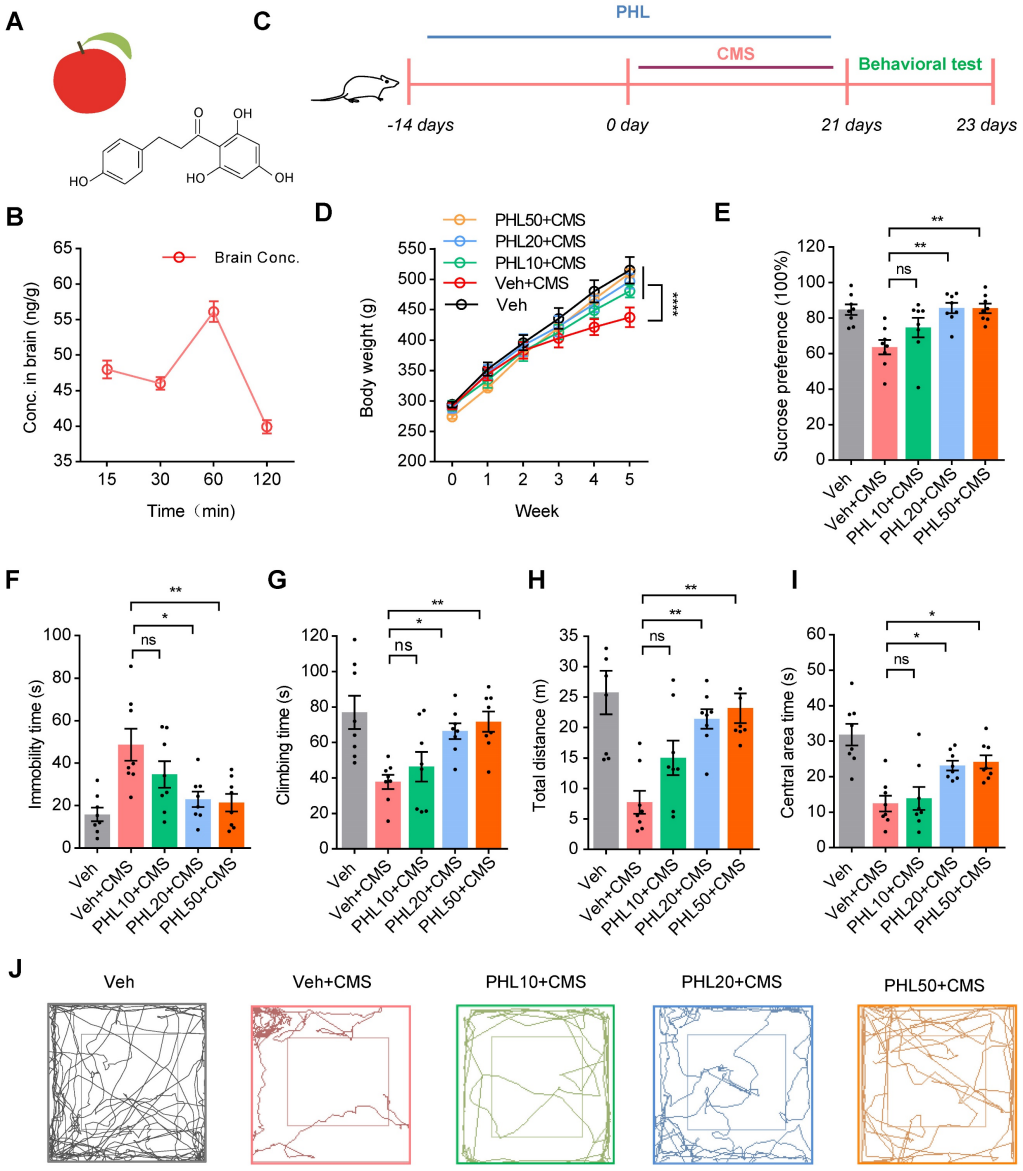
** PHL attenuates the CMS-induced depressive-like behaviors**.** (A)** The chemical structure of PHL. **(B)** Time concentration (Conc.) curve of PHL in the rat brain tissues (n = 3 rats per time point). **(C)** Schematic diagrams of the PHL and CMS treatment and behavioral tests.** (D)** Effects of different doses of PHL (10, 20, and 50 mg/kg) on the body weight of rats in different groups (n = 8 rats per group). **(E)** Sucrose preference was measured in CMS rats after PHL treatment (n = 8 rats per group).** (F-G)** The FST was evaluated in CMS rats after PHL treatment (n = 8 rats per group). The immobility time spent in the FST (**F**). The climbing time spent in the FST (**G**). **(H-I)** The OFT was performed in CMS rats after PHL treatment (n = 8 per group). The total distance traveled in the open field area (**H**). Time spent in the center (**I**). **(J)** Representative animal traces of rat movement in the OFT. Data are expressed as mean ± SEM. Two-way ANOVA with the Tukey's post hoc test (**D**). One-way ANOVA with the Tukey's post hoc test (**E-I**).^ ∗^*p* < 0.05, ^∗∗^*p* < 0.01, ^∗∗∗∗^*p* < 0.0001. ns, no significant difference.

PHL is a dihydrochalcone isolated from apples and apple-derived products (Figure 1A). To determine whether PHL could cross the rat blood-brain barrier (BBB), the level of PHL in the brain was determined by a liquid chromatography-tandem mass spectrometry (LC-MS/MS) following the intragastrical administration of 50 mg/kg PHL. We found that the peak level of PHL was detected in the brain at 60 min (Figure 1B), suggesting that PHL can cross the rat blood-brain barrier (BBB), which was consistent with a previous report in mice 25.

To determine whether PHL could display anti-depression effects, rats were received vehicle (0.5% CMC-Na) or PHL at the dosages of 10, 20, and 50 mg/kg for 2 weeks. Then, rats were exposed to CMS together with different dosages of PHL mentioned above for 3 week (Figure 1C). We found that PHL at the dosages of 10, 20, and 50 mg/kg obviously increased the body weight compared with Veh + CMS group (Figure 1D). Meanwhile, we demonstrated that 20 and 50 mg/kg of PHL efficiently improved the depression-like behaviors in the SPT, FST and OFT of CMS rats, whereas the dosage of 10 mg/kg of PHL exhibited no effects on the depression-like behaviors compared with Veh + CMS group (Figure 1E-I). In the OFT, the representative animal traces of different group rats were shown in Figure 1J. In addition, we also examined the motor ability with beam-balance test and prehensile traction test in different group rats. We found that there were no significant changes among them (Table S3).

To exclude the potential influence of single vehicle (CMC-Na) or PHL on the normal rat behaviors in the SPT, FST, and OFT, rats were received with CMC-Na or 20 mg/Kg of PHL for 5 weeks. We found that CMC-Na or PHL had no influences on rat behaviors (Figure S1-S2). In addition, we determined the effects of pre-treatment time of PHL on the depression-like behaviors, and found that 0, 1, or 7-day of pre-treatment of PHL did not significantly improve the depression-like behaviors (Figure S3). Therefore, the dosage of 20 mg/kg of PHL and the time of 2-week of PHL pre-treatment were selected for subsequent* in vivo* studies.

Given that PHL significantly increased the body weight compare with Veh + CMS group, we also analyzed the food intake in different group rats. We showed that the food intake of Veh + CMS group was lower than that of Veh group, whereas the food intake of PHL + CMS group was higher than that of Veh + CMS group. In addition, we found that there were no changes in the food intake between Veh group and PHL group (Table S4). Our data indicates that PHL not only influences the depression-like behaviors of CMS rats but also affects their food intake.

### PHL improves the neuronal structure in the mPFC after CMS exposure

**Figure 2:**
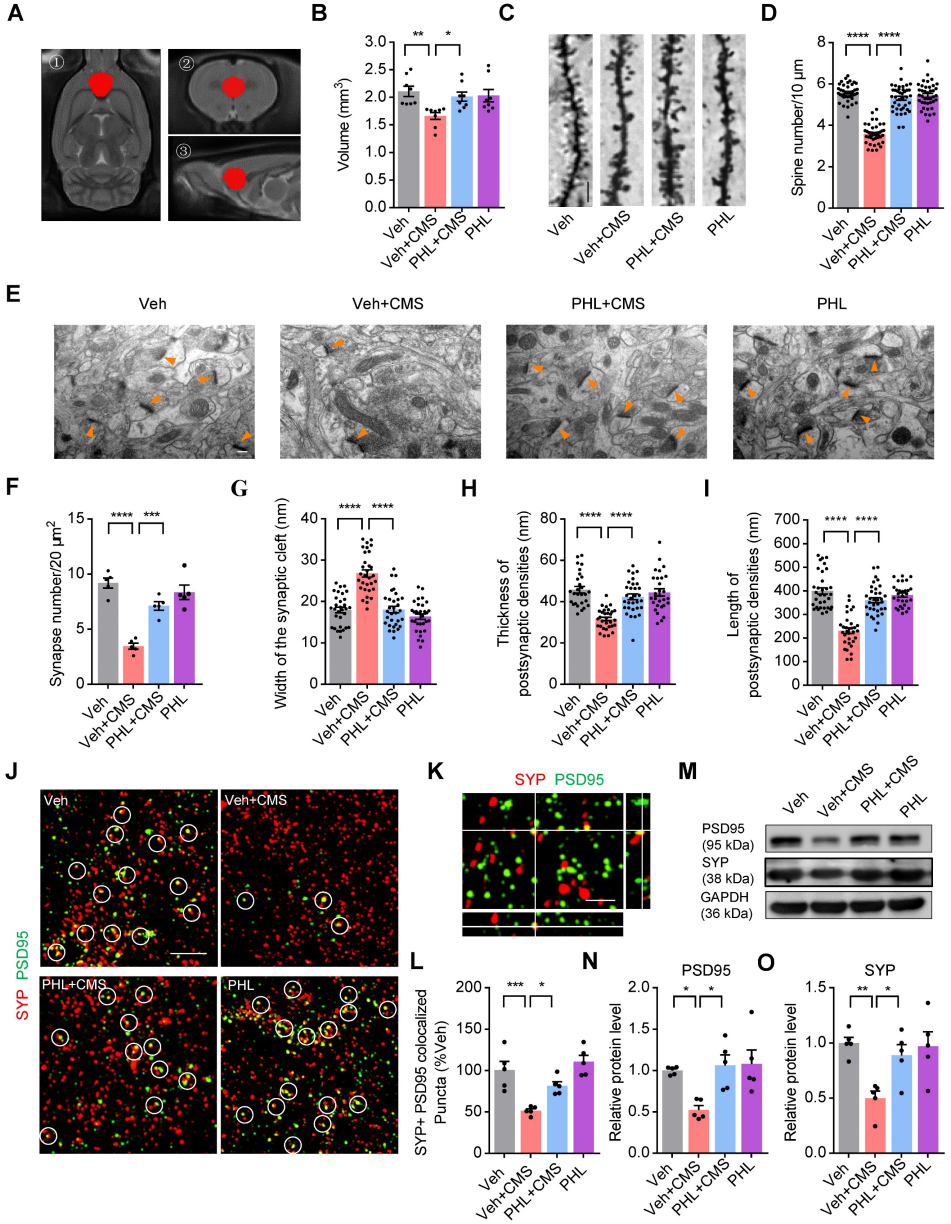
** PHL improves the neuronal structure in the mPFC after CMS exposure**.** (A)** The spatial location of the mPFC in the MRI. ① The axial view of the mPFC in T2 image. ② The coronal view of the mPFC in T2 image. ③ The sagittal view of the mPFC in T2 image. **(B)** Quantification of the volume of the mPFC in different group of rats (n = 8 rats per group). **(C)** The representative images of dendritic spine of pyramidal neurons in the mPFC by Golgi-Cox staining. Scale bar = 5 μm. **(D)** Quantification of dendritic spine density of pyramidal neurons in the mPFC (n = 40 neurons from 5 rats per group). **(E)** The representative electron micrographs of the mPFC sections. Orange arrows show synapses. Scale bar = 200 nm. **(F-I)** The number of synapse per 20 μm^2^ (**F**) (n = 5 rats per group), synaptic cleft width (**G**), thickness (**H**) and length (**I**) of postsynaptic density (PSD) were calculated by the Image J software (n = 27 to 34 synapses from 5 rats per group). **(J)** Super-resolution SIM images of Synaptophysin (SYP) (prestsynaptic marker) (red) and PSD95 (postsynaptic marker) (green) puncta in the mPFC. Co-localized puncta were indicated by circles. Scale bar = 4 μm. **(K)** Orthogonal view of SIM images showing co-localization of SYP (red) and PSD95 (green). Scale bar = 2 μm. **(L)** Quantification of the co-localization of SYP and PSD95 on super-resolution SIM images of the mPFC in different group of rats (n = 5 rats per group). **(M)** The expression of PSD95 and SYP in the mPFC of different group rats was detected by western blot. The GAPDH was served as the internal control. **(N-O)** Relative quantitative analysis of PSD95 and SYP protein level in the mPFC (n = 5 rats per group). Data are expressed as mean ± SEM. One-way ANOVA with Tukey's post hoc test (**B, D, F**-**I, L, N-O**). ^∗^*p* < 0.05, ^∗∗^*p* < 0.01,^ ∗∗∗^*p* < 0.001,^ ∗∗∗∗^*p* < 0.0001.

The neurons of the mPFC region are highly sensitive to stress 15. To determine whether PHL displays protective effects on the structure of the mPFC, we detected the volume change of mPFC after CMS exposure with MRI (Figure 2A). We found that CMS exposure obviously decreased the volume of mPFC compared with Veh group. However, the volume of mPFC was increased after the treatment of PHL in CMS rats (Figure 2B). Next, we examined the spine density changes in the mPFC by using Golgi Staining. The results from Golgi Staining demonstrated that CMS exposure significantly reduced dendritic spine density in the mPFC compared with Veh group, whereas the treatment of PHL remarkably increased the dendritic spine density compared with Veh + CMS group (Figure 2C-D). Moreover, we used ultra-structure transmission electron microscopy to examine the changes in synapse number and characteristic after CMS exposure. We found that the number of synapses, the cleft width of synapses, and the thickness and length of postsynaptic density were decreased in the mPFC after CMS exposure, whereas PHL improved these impairments compared with Veh + CMS group (Figure 2E-I).

Synapse loss is an important feature of stress-induced psychiatric disorders 13, we therefore examined whether there exists synapse loss in the mPFC after CMS exposure. Our SIM image showed that Synaptophysin (pre-synaptic puncta) and PSD95 (post-synaptic puncta) could co-localize in the mPFC (Figure 2K). Through quantification of the co-localized Synaptophysin and PSD95, we found that there was a significant loss of synapse in the mPFC after CMS exposure, whereas PHL repressed above losses compared with Veh + CMS group (Figure 2J and L). To determine whether the treatment of PHL could influence the level of PSD95 and synaptophysin (SYP) in the mPFC tissues of CMS rats, we carried out western blot analysis. We found that 20 μg of total protein was in the linear range for each target protein (Figure S4). In addition, we also showed that PHL remarkably improved the level of PSD95 and SYP compared with Veh + CMS group (Figure 2M-O).

### PHL improves the neuronal activity in the mPFC after CMS exposure

**Figure 3:**
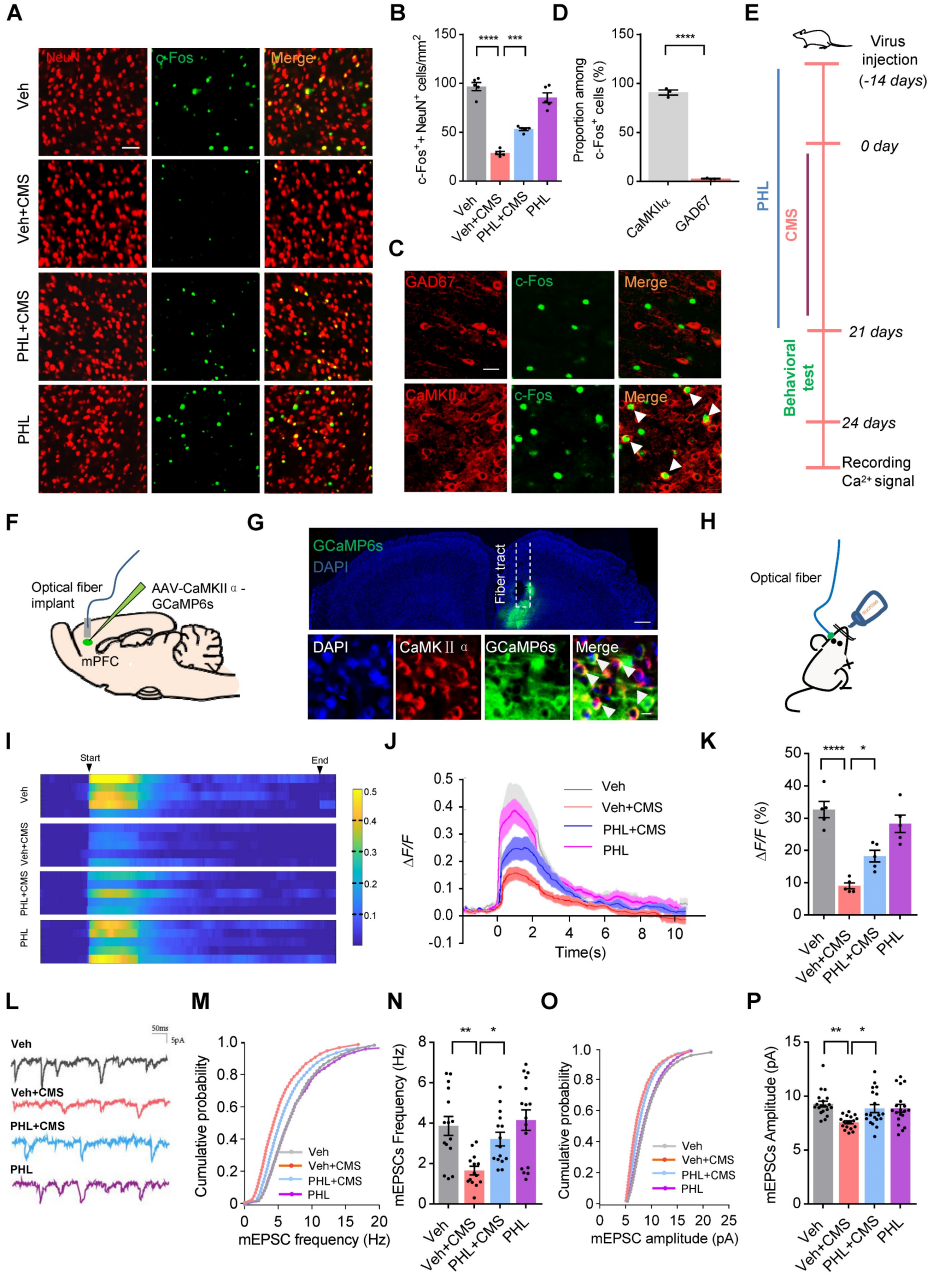
** PHL improves the neuronal activity in mPFC after CMS exposure**. **(A)** Representative immunostaining images of NeuN (red) and c-Fos (green) in the mPFC. Scale bar = 50 μm. **(B)** Quantification of c-Fos and NeuN double-positive cells in the mPFC (n = 5 rats per group). **(C)** Representative images of CaMKIIα or GAD67 positive cells co-labeled with c-Fos positive cells in the mPFC of Veh group rats after FST. White arrowheads showed the co-labeled cells. Scale bar = 20 μm. **(D)** Quantification of CaMKIIα or GAD67 and c-Fos double-positive cells in the mPFC of Veh group rats after FST (n = 3 rats per group). **(E)** Timeline of the experimental procedure for recording Ca^2+^ signals.** (F)** Schematic illustration of fiber photometry recording paradigm *in vivo*. **(G)** Representative images of the expression of GCaMP6s in excitatory neurons and the position of optical fiber tract in the mPFC (top). Scale bar = 200 μm. Bottom: representative images of the co-localization of GCaMP6s-expressing cells (green) and CaMKIIα positive cell (red) in the mPFC. White arrowheads showed the co-labeled cells. Scale bar = 20 μm. **(H)** Schematic of fiber photometry recording in the sucrose water intake-induced changes of Ca^2+^ signals. **(I)** Representative heat maps showed the changes of Ca^2+^ signals evoked by sucrose water in different groups. **(J-K)** Representative traces (**J**) and average ΔF/F (**K**) of GCaMP6s signals during in sucrose water intake (n = 5 rats per group). **(L)** Representative mEPSCs (mini excitatory postsynaptic current) traces from pyramidal neurons in the mPFC of different group rats. **(M-P)** Cumulative distribution (**M, O**) and average frequency (**N**) and amplitude (**P**) of mEPSCs in the mPFC of different group rats (n = 14 to 19 cells from 5 rats per group). Data are shown as mean ± SEM. One-way ANOVA with the Tukey's post hoc test (**B**, **K**,** N**, **P**). Two-tailed t tests (**D**). ^∗^*p* < 0.05, ^∗∗^*p* < 0.01,^ ∗∗∗^*p* < 0.001, ^∗∗∗∗^*p* < 0.0001.

It is well known that the mPFC is implicated in the regulation of maladapted emotional states, including depression 11. We therefore evaluated the neuronal activity by c-Fos immunostainings in the mPFC after FST in CMS rats. As shown in Figure 3A-B, fewer c-Fos^+^ neurons (co-localized with NeuN) were observed in the mPFC of CMS rats after FST compared with Veh group. However, PHL remarkably augmented the number of c-Fos^+^ neurons compared with Veh + CMS group. To further identify which kind of neuron was mainly activated after FST, CaMKIIα (an excitatory neuronal marker) or GAD67 (an inhibitory neuronal marker) was co-stained with c-Fos antibody in Veh group, respectively. Our data showed that c-Fos was predominately co-localized with CaMKIIα but little co-localized with GAD67 (Figure 3C-D). Furthermore, we also examined the number of c-Fos^+^ excitatory neurons (co-localized with CaMKIIα) in the mPFC of CMS rats after FST. We found that fewer c-Fos^+^ excitatory neurons were present in the mPFC of CMS rats compared with Veh group, whereas PHL efficiently increased the number of c-Fos^+^ excitatory neurons in the mPFC compared with Veh + CMS group (Figure S5A-B). All the above data suggested that CMS exposure obviously decreased the activity of excitatory neurons in the mPFC, whereas PHL efficiently improved above neuron activities.

To further confirm the above findings, we recorded the Ca^2+^ signals in the rat mPFC. The timeline of the experimental procedure for recording Ca^2+^ signals was shown in Figure 3E. We first expressed a genetically encoded calcium sensor (GCaMP6s) in excitatory neurons of the mPFC (Figure 3F-G), and found that these rats received GCaMP6s virus displayed depression-like behaviors after 3-week of CMS (Figure S6). Next, we used fiber photometry to monitor the sucrose water intake-induced changes of Ca^2+^ signals in excitatory neurons of the rat mPFC (Figure 3H). We found that the intensity of Ca^2+^ signals in the mPFC was remarkably lower in Veh + CMS group than that in Veh group, whereas PHL improved these deficits compared with Veh + CMS group (Figure 3I-K).

It has been known that stress causes pathological changes in synaptic plasticity of mPFC 15. Therefore, whole-cell patch-clamp recordings were carried out to examine the effect of PHL on the mEPSC in the mPFC of CMS rats. Our results showed that the frequency and amplitude of mEPSC were decreased after CMS exposure, while the treatment of PHL increased the frequency and amplitude of mEPSC in CMS rats (Figure 3L-P). In addition, we examined the effects of PHL on the induction of LTP and LTD in the mPFC of CMS rats, respectively. We found that the induction of LTP was decreased in CMS rats compared with Veh group, whereas the treatment of PHL increased it in CMS rats (Figure S5C-D). Meanwhile, we showed that the induction of LTD was increased in the mPFC of CMS rats compared with Veh group, whereas the treatment of PHL decreased it in CMS rats (Figure S5E-F). These results further suggest that PHL improves the neuronal activity in the mPFC after CMS exposure.

### PHL suppresses the microglia activation in the mPFC after CMS exposure

**Figure 4:**
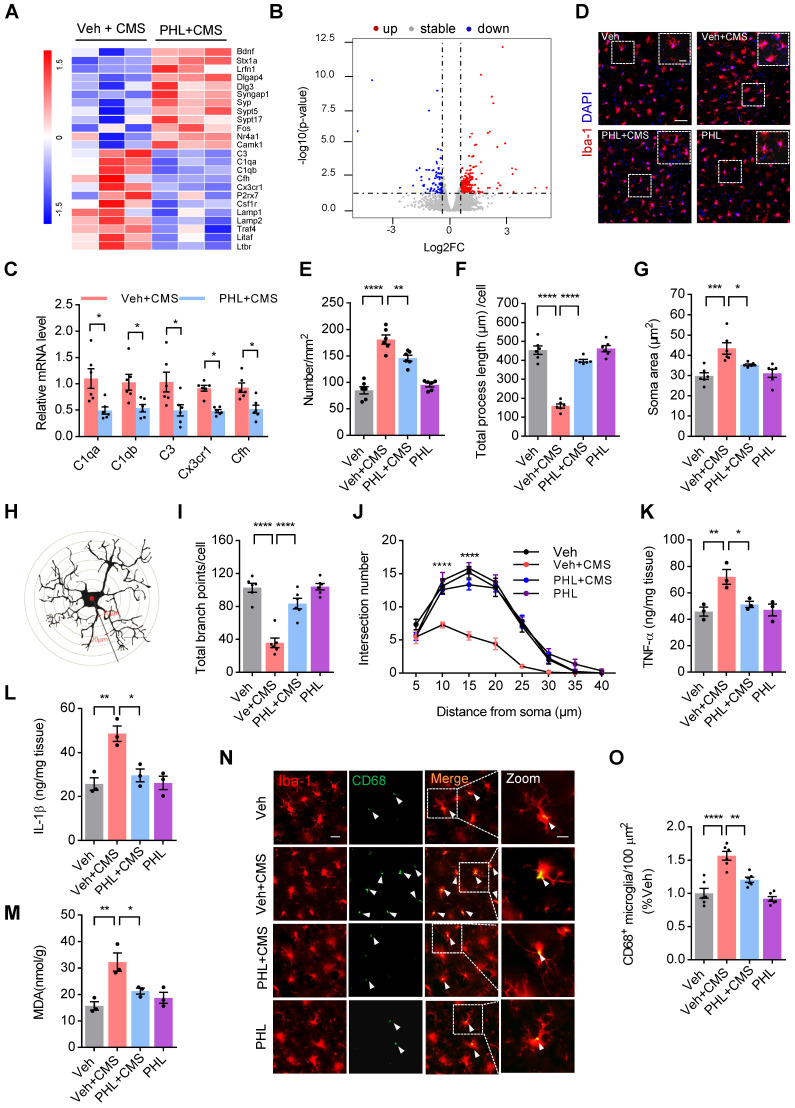
** PHL suppresses the microglia activation in the mPFC after CMS exposure. (A)** Heat maps of the differentially expressed genes determined by RNA-seq from the mPFC tissues. **(B)** Volcano plot showed the significantly differentiated genes. Up-regulated genes were shown in red, while down-regulated genes were shown in blue.** (C)** Real-time PCR was carried out to verify several candidate genes indicated in (**A**) after the treatment of PHL. **(D)** Representative immunostaining images of Iba-1^+^ positive microglia (Red) in the mPFC of different groups. Scale bar = 50 μm. Zoom in images (bar = 20 μm). **(E-G)** The histogram represents the quantification of number (**E**), process length (**F**), and soma area (**G**) of Iba-1^+^ cells in the mPFC of different groups (n = 6 rats per group). **(H)** The diagram of concentric Sholl radii for analyzing the morphology of microglia. **(I-J)** The number of total branch point per cell (**I**) and intersections per radius (**J**) were identified in different groups (n = 6 rats per group). **(K-M)** The levels of TNF-α (**K**), IL-1β (**L**) and MDA (**M**) in the mPFC tissues were measured in different groups (n = 3 rats per group). **(N)** Representative immunostaining images of Iba-1 (red) and CD68 (green) in the mPFC of different groups. Scale bar = 20 μm. Zoom in images (bar = 10 μm). **(O)** The CD68^+^ microglia were presented in different group (n = 6 rats per group). Data are shown as mean ± SEM. Two-tailed t tests (**C**). Two-way ANOVA with the Tukey's post hoc test (**J**). One-way ANOVA with the Tukey's post hoc test (**E**-**G**,** I**, **K-M**).^ ∗^*p* < 0.05, ^∗∗^*p* < 0.01,^ ∗∗∗^*p* < 0.001,^ ∗∗∗∗^*p* < 0.0001.

Considering that PHL exhibits an anti-depression effect, we explored the differentially expressed genes (DEGs) in the mPFC tissues between PHL + CMS group and Veh + CMS group by RNA sequencing. As shown in Figure 4A-B, the gene expression profile was changed by the treatment of PHL. Among those genes, we verified that PHL treatment decreased the level of complement cascade genes, such as C3, C1qa, C1qb, and Cfh. Meanwhile, we also showed that Cx3cr1, which is almost exclusively expressed in microglia, was reduced in the PHL+ CMS group compared with Veh + CMS group (Figure 4C). In addition, we examined the DEGs in the mPFC tissues between Veh group and Veh + CMS group and found that the expression of complement cascade genes, such as C3, C1qa, C1qb, and Cfh were increased after CMS exposure (Figure S7).

Next, the Kyoto Encyclopedia of Genes and Genomes (KEGG) pathway enrichment analyses were carried out to determine the functions and pathways of DEGs. KEGG enrichment analyses revealed that the treatment of PHL changed the expression of genes associated with the apelin signaling pathway, phagosome, lysosome, synaptic vesicle cycle, complement and coagulation cascades, and NF-kappa B pathway (Figure S8A). Meanwhile, we also performed gene set enrichment analyses (GSEAs) and found that PHL influenced the expression of genes in the processes of microglial cell activation and structural constituent of synapse (Figure S8B). Above findings suggest that PHL might influence the microglia-mediated phagocytosis in the mPFC.

It is well documented that stress can activate microglia and in turn induce morphological changes, phagocytosis, and release of pro-inflammatory cytokines 26, 27. To evaluate the effect of PHL on the activation of microglia, we examined the number of microglia in the mPFC by using Iba-1 (microglia marker) antibody after 3-week of CMS exposure. The number of Iba-1^+^ microglia in the mPFC was increased after 3-week of CMS exposure, while PHL inhibited this increase (Figure 4D-E). We also investigated the morphological changes of microglia after 3-week of CMS exposure in the mPFC and presented a representative diagram of microglia for sholl analysis in Figure 4H. We found that 3-week of CMS exposure induced a larger soma area, but a shorter length of microglia processes and a decreased branch point number and intersection number in the mPFC, whereas PHL significantly repressed these changes (Figure 4F-G, I-J). In addition, we detected the level of tumor necrosis factor-α (TNF-α), interleukin-1β (IL-1β), and malondialdehyde (MDA), which are often linked with microglia activation in the mPFC. We showed that the level of TNF-α, IL-1β, and MDA were obviously increased in the mPFC of CMS rats, whereas these changes were significantly prevented by the treatment of PHL (Figure 4K-M).

CD68 is a lysosomal marker that can reflect the microglial phagocytic activity. To analyze the microglial phagocytic activity induced by CMS exposure, we examined the number of CD68^+^ (green) per Iba-1^+^ (red) microglia in CMS rats. We showed that CMS exposure increased the number of CD68 positive microglia in the mPFC compared with Veh group, whereas PHL decreased these increases (Figure 4N-O), indicating that PHL can decrease the microglial phagocytic activity after CMS exposure.

### Microglia mediated synaptic engulfment is involved in the CMS-induced depression-like behaviors in the mPFC

**Figure 5:**
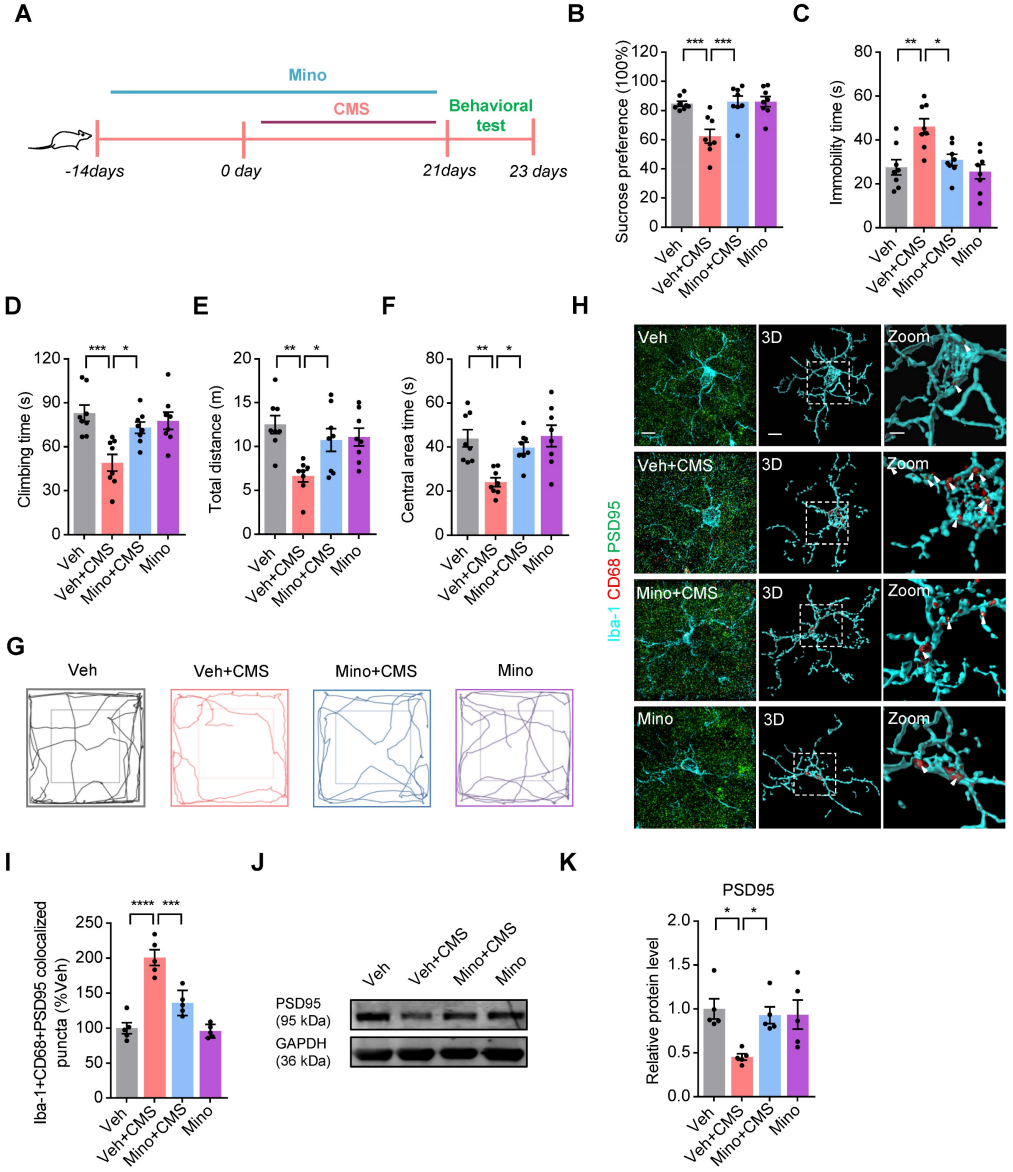
**Microglia mediated synaptic engulfment is involved in the CMS-induced depression-like behaviors in the mPFC. (A)** Schematic diagrams of the Minocycline (Mino) and CMS treatment and behavioral tests. **(B)** Sucrose preference was measured in CMS rats after Mino treatment (n = 8 rats per group).** (C-D)** The FST was evaluated in CMS rats after Mino treatment (n = 8 rats per group). The immobility time spent in the FST (**C**). The climbing time spent in the FST (**D**). **(E-F)** The OFT was performed in CMS rats after Mino treatment (n = 8 per group). The total distance traveled in the open field area (**E**). Time spent in the center (**F**). **(G)** Representative animal traces of rat movement in the OFT. **(H)** Representative images and 3D reconstruction rendering of Iba-1^+^ microglia (cyan) containing PSD95^+^ puncta (green) and CD68 (red). Scale bar = 5μm. Zoom in images (bar = 2 μm). White arrowheads showed the co-localization between PSD95 and CD68 in microglia. **(I)** Quantification of the co-localization of PSD95 and CD68 in Iba-1^+^ microglia after the treatment of Mino (n = 5 rats per group). **(J)** The expression of PSD95 in the mPFC of different group rats was detected by western blot. The GAPDH was served as the internal control.** (K)** Relative quantitative analysis of PSD95 protein level in the mPFC (n = 5 rats per group). Data are shown as mean ± SEM. One-way ANOVA with the Tukey's post hoc test (**B-F, I, K**). ^∗^*p* < 0.05, ^∗∗^*p* < 0.01, ^∗∗∗^*p* < 0.001, ^∗∗∗∗^*p* < 0.0001.

Given that CMS exposure increases microglial phagocytic activities and synapse losses in the mPFC, we examined whether microglia mediated synaptic engulfment is involved in the CMS-induced depression-like behaviors. To determine this possibility, we used minocycline to block the microglia activation and phagocytosis in CMS rats, and the timeline of the experimental procedure was shown in Figure 5A. We found that minocycline repressed the CMS-induced microglia activation and phagocytosis in CMS rats (Figure S9). Meanwhile, we also showed that minocycline efficiently improved the depression-like behaviors in the SPT, FST and OFT of CMS rats (Figure 5B-G). The PSD95^+^ puncta has been documented as a synaptic element in the studies of microglia-mediated synaptic engulfment 28. Then, we investigated the co-localization of PSD95^+^ puncta with CD68 in microglia after CMS exposure in the mPFC. We showed that there was a relatively high percentage of PSD95 co-localized with CD68 in microglia after CMS exposure, whereas minocycline decreased these co-localizations (Figure 5H-I and Video S1-S4). In addition, we also examined the expression of PSD95 in the mFPC after the treatment of minocycline in CMS rats, and found that minocycline increased the level of PSD95 in the mFPC of CMS rats (Figure 5J-K). All these data suggests that microglia mediated synaptic engulfment is involved in the CMS-induced depression-like behaviors.

### PHL decreases the CMS-induced synapse losses by inhibiting the deposition of C3 on synapses and subsequent microglia-mediated synaptic engulfment in the mPFC

**Figure 6:**
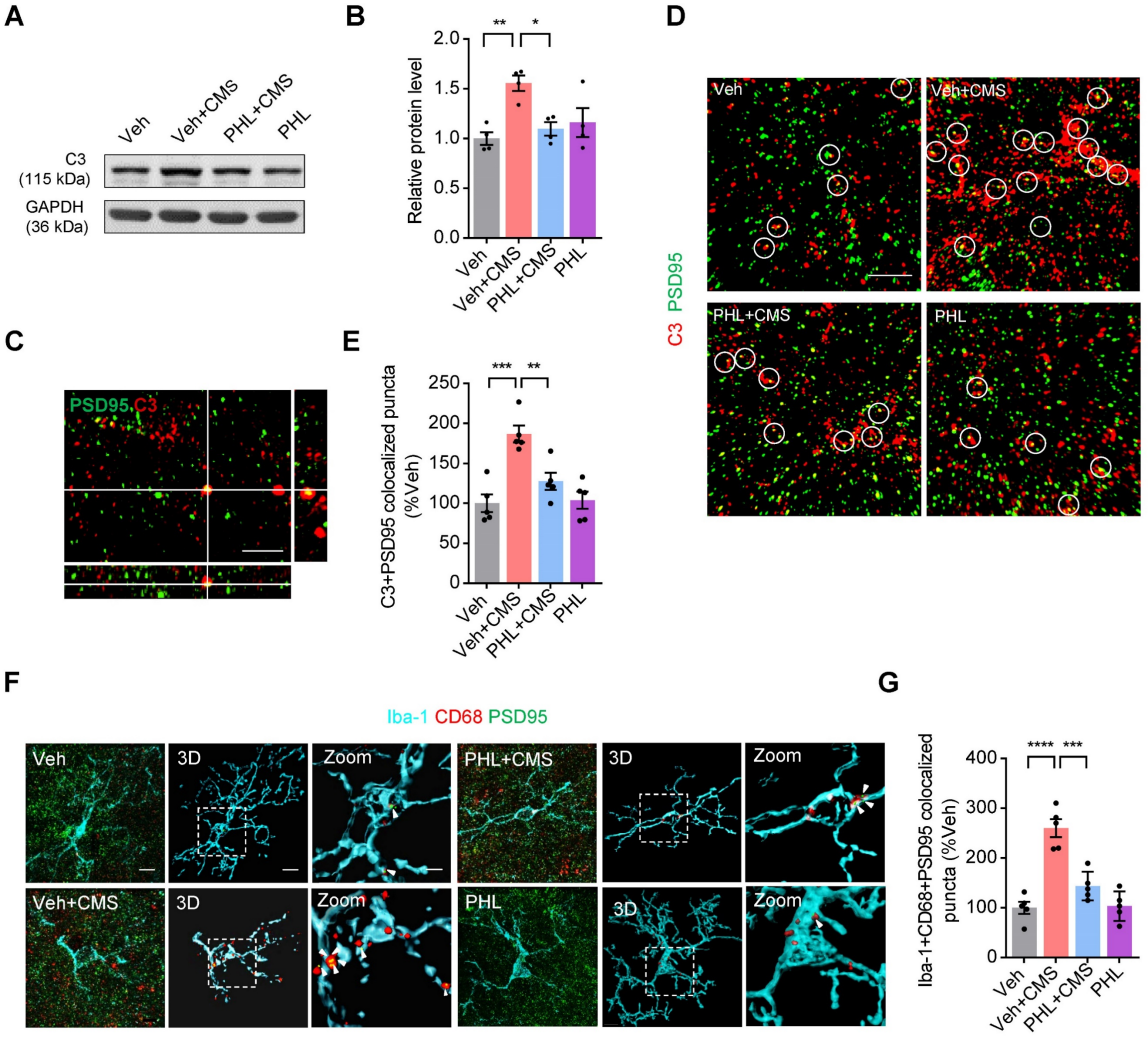
** PHL decreases the CMS-induced C3 deposition onto synapses and subsequent synaptic engulfment in the mPFC**. **(A)** The expression of C3 in the mPFC was detected by western blot. The GAPDH was served as the internal control. **(B)** Relative quantitative analysis of C3 protein level in the mPFC (n = 4 rats per group).** (C)** Orthogonal view of SIM images showed co-localization of C3 (red) and PSD95 (green). Scale bar = 2 μm. **(D)** Representative immunostaining images showed higher percentage of PSD95 (green) co-localized with C3 (red) in CMS rats. Co-localized puncta were indicated by circles. Scale bar = 4 μm. **(E)** Quantification of the co-localization of PSD95 and C3 in the mPFC of CMS rats after the treatment of PHL (n = 5 rats per group). **(F)** Representative images and 3D reconstruction rendering of Iba-1^+^ microglia (cyan) containing PSD95^+^ puncta (green) and CD68 (red). Scale bar = 5μm. Zoom in images (bar = 2 μm). White arrowheads showed the co-localization between PSD95 and CD68 in microglia. **(G)** Quantification of the co-localization of PSD95 and CD68 in Iba-1^+^ microglia after the treatment of PHL (n = 5 rats per group). Data are shown as mean ± SEM. One-way ANOVA with the Tukey's post hoc test (**B, E, G**). ^∗^*p* < 0.05, ^∗∗^*p* < 0.01,^ ∗∗∗^*p* < 0.001, ^∗∗∗∗^*p* < 0.0001.

It has been shown that complement molecules C3 and C1q localize to synapses and mediate synapse losses in neurodegenerative diseases by phagocytic microglia 17, 28, 29. Since complement protein C3 is a central molecule in the complement system, we therefore speculate that C3 might also localize to synapse after CMS exposure. To this end, we first examined the expression of C3 in the mPFC after 3-week of CMS exposure. Our western blot data showed that the elevation of C3 levels was observed in the mPFC of CMS rats. However, PHL attenuated the CMS-induced increase of C3 in the mPFC (Figure 6A-B).

Next, we analyzed the co-localization between C3 and PSD95 in the mPFC after 3-week CMS exposure. Our SIM image showed that C3 and PSD95 could co-localize in the mPFC (Figure 6C). Meanwhile, we found that there was a higher percentage of PSD95^+^ puncta co-localized with C3 in the mPFC after CMS exposure. However, PHL remarkably reduced the deposition of C3 onto synapses (Figure 6D-E). Given that C3 is involved in the microglia-mediated synaptic engulfment, we investigated the co-localization of PSD95^+^ puncta with CD68 in microglia after the treatment of PHL in the mPFC. Our data showed that there was a relatively high percentage of PSD95 co-localized with CD68 in microglia after CMS exposure. However, PHL reduced these co-localizations (Figure 6F-G and Video S5-S8). All above data suggest that PHL decreases the CMS-induced synapse losses by inhibiting the deposition of C3 on synapses and subsequent microglia-mediated synaptic engulfment.

### PHL decreases the expression of C3 *via* inhibiting the NF-κB pathway

**Figure 7:**
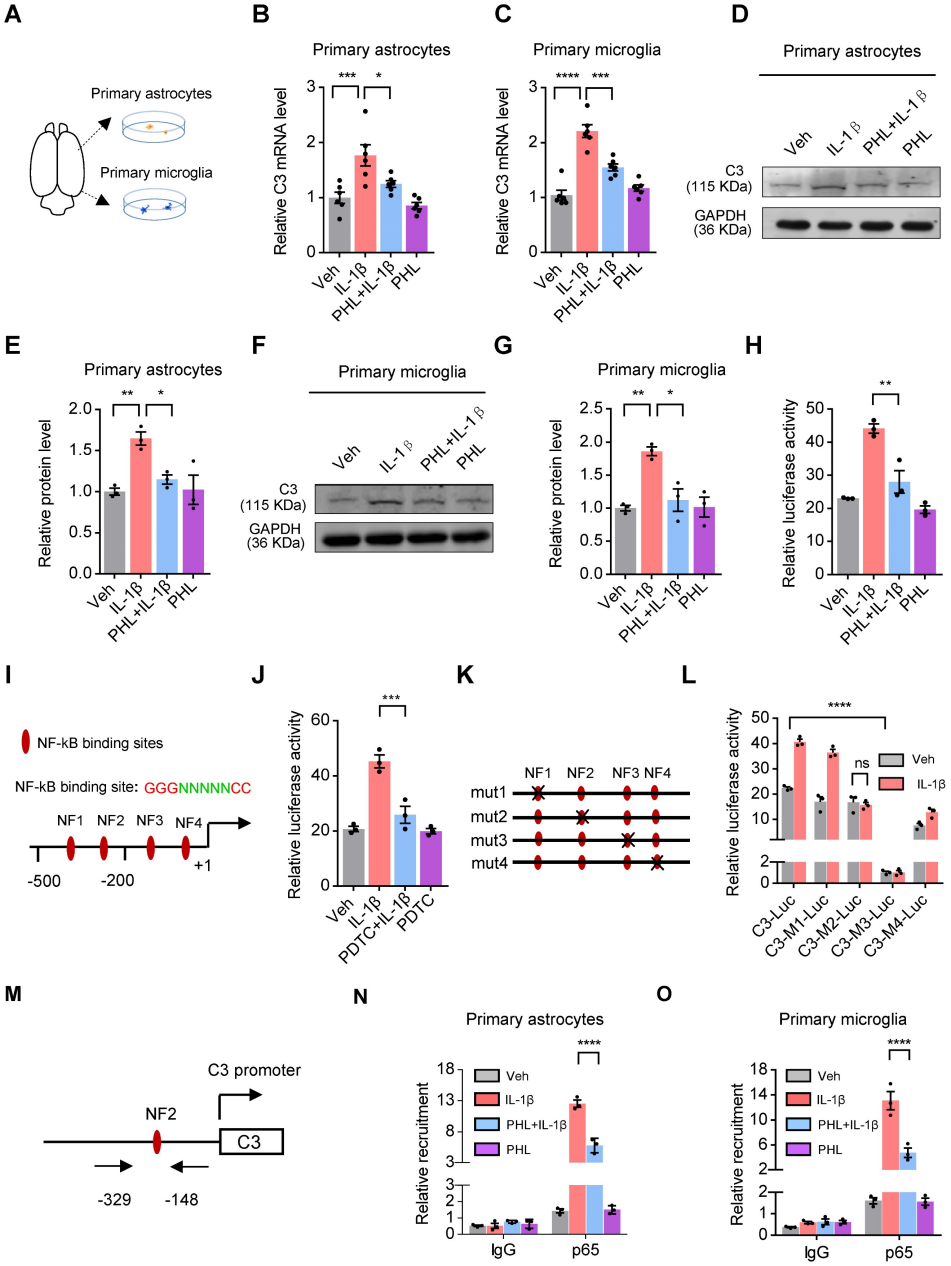
** PHL decreases the expression of C3 *via* inhibiting the NF-kB pathway**. **(A)** Schematic experimental procedure to prepare for rat primary cultured microglia and astrocytes. **(B-C)** The mRNA level of C3 was examined in rat primary astrocytes (**B**) and microglia (**C**) (n = 6 per group). (**D, F**) The protein level of C3 was detected by western blot in rat primary astrocytes (**D**) and microglia (**F**). **(E**, **G)** Relative quantitative analysis of C3 protein level in primary astrocytes (**E**) and microglia (**G**) (n = 3 per group). **(H)** Effects of PHL on the IL-1β-induced C3 promoter activity in BV2 cells (n = 3 per group). **(I)** Schematic structure of the rat C3 promoter and the core sequence of NF-κB binding site. The four putative NF-κB binding sites were named as NF1, NF2, NF3 and NF4, respectively. **(J)** Effects of PDTC on the IL-1β-induced C3 promoter activity in BV2 cells (n = 3 per group). **(K)** Schematic drawings of four mutated NF-κB binding site constructs. **(L)** Effects of mutation of NF-κB binding sites on the IL-1β-induced C3 promoter activity in BV2 cells (n = 3 per group). **(M)** The location of primers in the C3 promoter for ChIP assays was shown. **(N-O)** Effects of PHL on the DNA binding activity of p65 to the NF2 site of C3 promoter in primary rat astrocytes (**N**) and microglia (**O**) in the presence of IL-1β by ChIP assays (n = 3 per group). Data are shown as mean ± SEM. One-way ANOVA with the Tukey's post hoc test (**B, C, E, G, H, J**). Two-tailed t tests (**L**). Two-way ANOVA with the Tukey's post hoc test (**N-O**).^ ∗^*p* < 0.05, ^∗∗^*p* < 0.01,^ ∗∗∗^*p* < 0.001,^ ∗∗∗∗^*p* < 0.0001.

Microglia and astrocytes have been documented as the primary sources of C3 in primary cultures 30. IL-1β, a release product of activated microglia, has been shown to induce the expression of C3 in mouse astrocytes 31. To determine whether IL-1β could induce the expression of C3 in rat primary astrocytes and microglia, we prepared the rat primary astrocytes and microglia (Figure 7A) and examined the expression of C3 in the presence of IL-1β. Our results demonstrated that IL-1β induced the expression of C3 in rat primary microglia and astrocytes, whereas PHL significantly reduced the IL-1β-induced C3 expression (Figure 7B-G).

We next investigated the underlying mechanisms that PHL reduced the IL-1β-induced rat C3 expression. First, we examined whether IL-1β could activate the rat C3 promoter activity in BV2 cells. As we expected, IL-1β significantly increased the C3 promoter activity, while PHL efficiently decreased the IL-1β-induced the C3 promoter activity (Figure 7H). Second, using TRANSFAC database, we found four putative NF-κB sites in the upstream of rat C3 gene (Figure 7I). Considering that IL-1β could activate the NF-κB pathway 32, we examined the effects of NF-κB inhibitor PDTC on the C3 promoter activity in the presence of IL-1β. Our data showed that NF-κB inhibitor PDTC obviously decreased the IL-1β-induced C3 promoter activity (Figure 7J). To determine which of the above four putative NF-κB sites of the C3 promoter plays a crucial role in the regulation of C3 promoter activity, we mutated the above NF-κB sites one by one as shown in Figure 7K. Luciferease activities showed that mutation of NF3 site greatly reduced the activity of C3 promoter. Importantly, we found that mutation of NF2 site abolished the IL-1β-induced C3 promoter activity, suggesting the crucial role of NF2 site in the regulation of IL-1β-mediated C3 promoter activity (Figure 7L).

The p65 subunit of NF-κB is necessary for the activation of the NF-κB pathway 33. We then examined the effects of PHL on the phosphorylation of p65 and translocation of p65 to nuclear in rat primary microglia and astrocytes. We found that PHL efficiently reduced both the IL-1β-induced phosphorylation of p65 and the translocation of p65 to nuclear (Figure S10). Moreover, we investigated whether PHL could repress p65 binding to the NF2 site of the C3 promoter in the presence of IL-1β by ChIP assay. The schematic diagram of primer position for ChIP assay was shown in Figure 7M. Our ChIP data showed that IL-1β increased the binding capacity of p65 to the NF2 site of the C3 promoter. However, PHL significantly decreased this binding activity (Figure 7N-O). All these results suggest that PHL decreases the expression of C3 by inhibiting the NF-κB pathway.

### PHL represses the NF-κB-C3 axis to prevent the CMS-induced depressive-like behaviors in the mPFC

**Figure 8:**
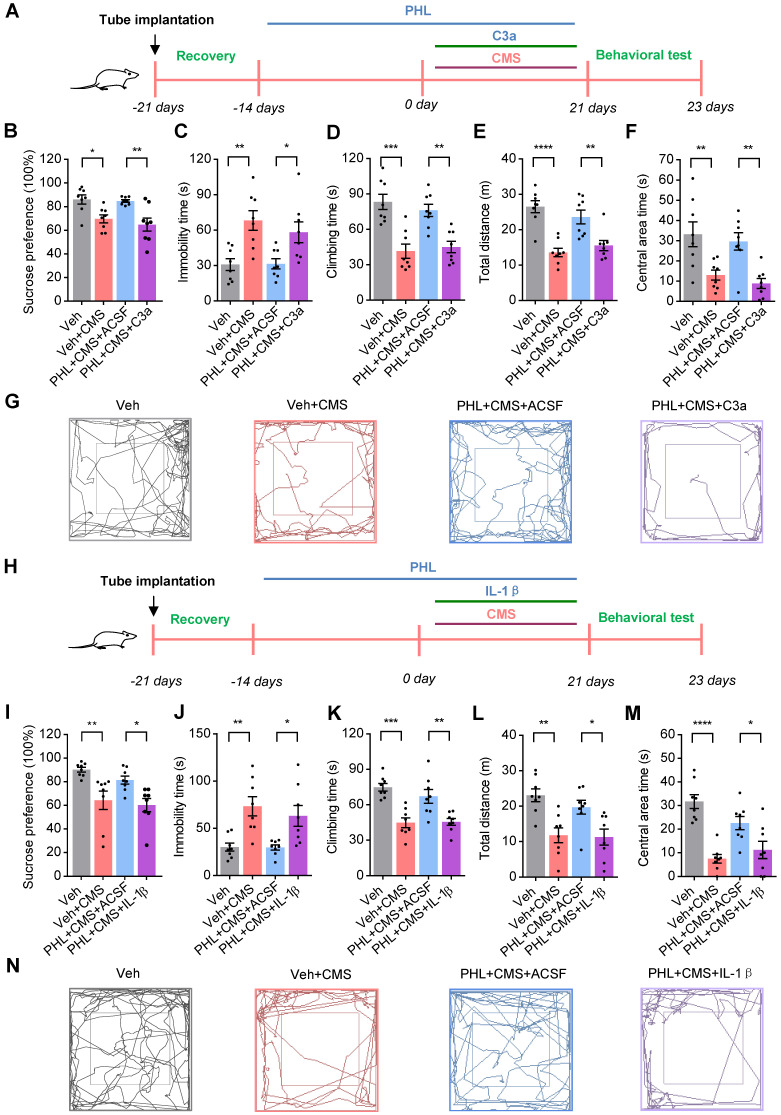
**PHL represses the NF-κB-C3 axis to prevent the CMS-induced depressive-like behaviors in the mPFC**. **(A)** Schematic diagrams of the C3a injection and behavioral tests. **(B)** Sucrose preference was measured after the injection of C3a (n = 8 rats per group).** (C-D)** The FST was evaluated after the injection of C3a (n = 8 rats per group). The immobility time spent in the FST (**C**). The climbing time spent in the FST (**D**). **(E-F)** The OFT was performed after the injection of C3a (n = 8 rats per group). The total distance traveled in the open field area (**E**). Time spent in the center (**F**). **(G)** Representative animal traces of rat movement in the OFT after the injection of C3a. **(H)** Schematic diagrams of the IL-1β injection and behavioral tests. **(I)** Same as in (**B**) but injection of IL-1β. **(J-K)** Same as in (**C-D**) but injection of IL-1β. **(L-M)** Same as in (**E-F**) but injection of IL-1β. **(N)** Representative animal traces of rat movement in the OFT after the injection of IL-1β. Data are shown as mean ± SEM. One-way ANOVA with the Tukey's post hoc test (**B-F, I-M**). ^∗^*p* < 0.05, ^∗∗^*p* < 0.01,^ ∗∗∗^*p* < 0.001, ^∗∗∗∗^*p* < 0.0001.

Since PHL represses the deposition of C3 on synapses and subsequent synapse losses, we investigated whether C3 could inhibit the anti-depression effect of PHL. To this end, C3a (70-77), an octapeptide of C3a (a cleavage product of C3), was injected into the mPFC of the PHL-treated rats with the cannula and rats were then exposed to 3-week of CMS treatment (Figure 8A). After 3-week CMS exposure, animal behaviors data showed that C3a (70-77) decreased the neuroprotective effect of PHL in the SPT, FST, and OFT (Figure 8B-G), suggesting that the anti-depression effect of PHL might be mediated by inhibiting the expression of C3.

To further determine whether PHL represses the NF-κB-C3 axis to exert the anti-depression effect, the IL-1β was adopted to activate the NF-κB pathway. We then injected IL-1β into the mPFC of the PHL-treated rats and treated rats with 3-week of CMS (Figure 8H). After 3-week CMS exposure, our data showed that IL-1β reduced the anti-depression effect of PHL in OFT, SPT, and FST (Figure 8I-N). Meanwhile, IL-1β increased the level phosphorylation of p65 (p-p65) and C3 in the mPFC compared with the PHL+ACSF+CMS group (Figure S11). However, the NF-κB pathway inhibitor PDTC not only improved the depression-like behaviors but also decreased the level of p-p65 and C3 in the mPFC after CMS exposure (Figure S12). All above results indicate that PHL represses the NF-κB-C3 axis to display the anti-depression effect in the mPFC.

## Discussion

In the current study, we showed that PHL protected against CMS-induced depression-like behaviors in rats. We also found that PHL repressed the decrease of synapse losses and neuronal activity in mPFC after CMS exposure. Furthermore, we showed that PHL decreased the microglial activation and phagocytic activity after CMS exposure. Importantly, we demonstrated that PHL repressed the NF-κB-C3 axis and subsequent microglia-mediated synaptic engulfment to prevent the CMS-induced depressive-like behaviors in the mPFC.

Stress, as an important risk factor for psychiatric disorders, has been reported to induce neuroinflammation and oxidative stress in the brain 34. Given that PHL has anti-inflammatory and anti-oxidative effects 19, it raises the question of whether PHL could display anti-depression effects. Indeed, we found that 2-week of pre-treatment of PHL efficiently improved the depression-like behaviors induced by 3-week of CMS exposure, whereas 0, 1, or 7-day of pre-treatment of PHL exhibited little effects, suggesting that the generation of protective effects of PHL needs sufficient time in CMS rats. We speculate that sufficient treatment time of PHL could maintain the normal expression of synaptic proteins and the number of synapses, which keep rats entering standby mode to cope with ongoing environmental stress.

Increasing evidences have shown that stress can change neuronal plasticity 2, 10, 35. Here, we showed that CMS exposure changed the dendritic complexity and synapse numbers in the mPFC, which was consistent with the previous findings 35. It has been reported that the volume of the left subgenual prefrontal cortex is reduced in depression 36. Similar to the previous findings, we found that CMS exposure decreased the volume of mPFC. We speculated that this decrease might partially be due to the changes in dendritic complexity and synapse number. Altered neuronal activity in the mPFC has been observed in social defeat stress mice 16. Here, we demonstrated that the activity of neurons in the mPFC response to FST and sucrose water was decreased after CMS exposure. Moreover, we also showed that CMS decreased the induction of LTP but facilitated the induction of LTD in CMS rats compared with Veh group. Our data suggest that the abnormal neuronal activity in mPFC might be present in several depression models. Polyphenolic compounds, such as green tea extracts and Curcumin, have been reported to modulate mental health by influencing brain plasticity 37. Here, we showed that PHL has similar protective effects. These protective effects observed in PHL might be mediated by increasing the spine density but decreasing the synapse losses in the mPFC like Curcumin 38.

Several studies have reported that stress can activate microglia in the pre-frontal cortex (PFC) and hippocampus (HPC) in mice 39. Here, we showed that 3-week of CMS treatment activated microglia in the mPFC of CMS rats. It is well known that activated microglia release pro-inflammatory mediators and oxidative products such as IL-1β, TNF-α, and MDA, which promote neuronal damage and induce the development of depression 26. Consistent with previous studies, we showed that the level of IL-1β, TNF-α and MDA were increased after 3-week of CMS, whereas PHL significantly inhibited the above increases. Interestingly, Curcumin has similar effects like PHL on the activation of microglia 40, further suggesting the protective role of polyphenolic compounds in abnormal activation of microglia.

Microglia have recently been recognized as a key regulator of synaptic connectivity *via* microglia mediated-synaptic elimination both in the healthy and diseased brain 17. A previous study has reported that minocycline can alleviate depression-like symptoms by blocking the microglia activation and phagocytosis in CMS mice 41. Here, we found that minocycline also displayed similar effects on CMS rats. Furthermore, we showed that minocycline repressed the microglia-mediated synaptic elimination in CMS rats. All these findings suggest that microglia mediated synaptic elimination is involved in the CMS-induced depression-like behaviors. In the developing mouse brain, Complement C1q and C3 target subsets of synapses and are necessary for synapse elimination by microglia 42, 43. Here, we showed that C3 also localized to synapses in the mPFC after CMS exposure like C1q localizing to synapses in Alzheimer's disease (AD) mouse model 28. Importantly, we found that the co-localization of PSD95 with CD68 was significantly decreased in Iba-1^+^ microglia of the mPFC after the treatment of PHL, suggesting the protective role of PHL in microglia-mediated synapse engulfment after CMS exposure.

Complement protein C3 is a central molecule in the complement system. Besides acting as an essential immune regulator, C3 also regulates the development of depression. It has been shown that the level of C3 is increased in postmortem brain of depressed subjects and stress-related model mice 44, 45. Here, we found that the level of C3 was increased in the mPFC of CMS rats, whereas PHL reduced this increase. Importantly, we showed that the injection of C3a in the mPFC reduced the protective effect of PHL after CMS exposure, suggesting PHL might display the anti-depression effects by inhibiting the expression of C3 in the mPFC. In the future, we will investigate whether PHL could regulate the expression of C3 receptors including C3aR or CR3. Meanwhile, we will determine whether inhibition of the expression of C3 receptors in the mPFC could improve the depression-like behaviors by decreasing CMS-induced synapses engulfment besides the mechanism of inhibiting inflammatory infiltration described by Crider et al. 45.

Considering that PHL can decrease IL-1β-induced C3 expression, we investigated the underlying mechanisms. We showed that four putative NF-κB binding sites are present in the rat C3 promoter. Meanwhile, NF-κB inhibitor PDTC efficiently reduced the activity of IL-1β-induced C3 promoter, confirming the role of IL-1β in regulation of C3 expression *via* the NF-κB pathway. Although we found four putative NF-κB binding sites in the C3 promoter, only mutation of the NF3 site greatly decreased the activity of the C3 promoter in the absence of IL-1β, indicating that NF3 site is responsible for the constitutive activity of C3 promoter. Interestingly, mutation of NF2 site abolished IL-1β-induced transcriptional activity, suggesting the crucial role of the NF2 site in IL-1β-induced transcriptional activity. We speculate that NF-κB had a higher binding activity to the NF2 site of the C3 promoter compared with the other three sites in the presence of IL-1β, and we will determine this possibility in the future.

The p65 subunit of NF-κB is necessary for the activation of the NF-κB pathway 33. In response to stimulation, p65 is phosphorylated and translocated from the cytoplasm into the nucleus to activate target gene expression 46, 47. Here, we found that IL-1β increased the level of p-p65 and the translocation of p65 to nuclear in rat primary microglia and astrocytes, whereas PHL repressed these increases. A recent study has shown that Astragalin, a flavonoid compound, reduced the DNA binding activity of NF-κB after the treatment of TNF-α 48. Here, we showed that PHL also decreased the DNA binding activity of NF-κB to the NF2 site of the C3 promoter in the presence of IL-1β, suggesting that PHL could repress the NF-κB pathway by inhibiting p65 binding to NF-κB site and in turn decrease the expression of C3.

It is well known that IL-1β induces depression-like behaviors by activating the NF-κB pathway 49. Here, we showed that the anti-depression effect of PHL was significantly attenuated by injection of IL-1β in the mPFC. However, we demonstrated that PTDC significantly attenuated the depression-like behaviors and the expression of C3 after CMS exposure in rats. Therefore, PHL might act as a natural NF-κB pathway inhibitor to decrease the NF-κB-mediated C3 expression, suggesting the potential role of PHL in the prevention of depression.

In conclusion, we demonstrate that PHL protects against CMS-induced depression behaviors. Moreover, we show that PHL inhibits the NF-κB-C3 axis to decrease the complement-mediated microglial synaptic engulfment in CMS-induced depression models. Our findings suggest that PHL might be a promising natural compound to prevent the emergence of stress-related psychiatric disorders in at-risk populations.

## Supplementary Material

Supplementary methods, figures, tables, video legends.

Supplementary video 1.

Supplementary video 2.

Supplementary video 3.

Supplementary video 4.

Supplementary video 5.

Supplementary video 6.

Supplementary video 7.

Supplementary video 8.

## Abbreviations

PHL: Phloretin
CMS: chronic mild stress
mPFC: medial prefrontal cortex
SPT: sucrose preference test
FST: forced swimming test
OFT: open field test
MRI: Magnetic Resonance Imaging
ACSF: artificial cerebrospinal fluid
FBS: fetal bovine serum
SYP: synaptophysin
PSD95: postsynaptic density 95
mEPSC: mini excitatory postsynaptic current
LTP: long term potentiation
LTD: Long term depression
ChIP: chromatin immunoprecipitation
PDTC: pyrrolidinedithiocarbamate ammonium
GAPDH: glyceraldehyde-3-phosphate dehydrogenase
