# Supplementary material for: Phloretin decreases microglia-mediated synaptic engulfment to prevent chronic mild stress-induced depression-like behaviors in the mPFC
Source: Theranostics. 2023 Jan 16;13(3):955–72. doi: 10.7150/thno.76553 (PMC9925308; doi:10.7150/thno.76553)
Supplement: Supplementary file 1 — Supplementary methods, figures, tables, video legends. [file thnov13p0955s1.pdf]

Supplementary Materials

Phloretin decreases microglia-mediated synaptic engulfment to prevent chronic mild stress-induced depression-like behaviors in the mPFC

Chenchen Li[1†], Bo Liu[1, 2†], Jingyi Xu[1†], Bin Jing[3†], Lijie Guo[1], Liyong Wang[4], Milin Wang[1], Haochen Zhang[1], Qianqian He[1], Xin Yu[1], Yan Zhang[5], Zhi-Qing David Xu[1, 6*], Yutao Yang[1*].

† These authors contributed equally to this work.

* Corresponding author.

This file includes:

Supplementary material and methods;

Supplementary tables: Table S1 to S4;

Supplementary figures and legends: Figure S1 to S12;

Supplementary video captions: Video S1 to S8;

Supplementary references;

Supplementary Materials and Methods

Chemicals and reagents

Phloretin was obtained from TCI (#P1966, TCI, Tokyo, Japan) and dissolved in 0.5% (w/v) CMC-Na (#C9481, Sigma-Aldrich, St. Louis, USA) solution. Complement C3a (70-77) was obtained from AnaSpec (#AS-61118, AnaSpec, San Jose, USA). Recombinant rat IL-1β was purchased from Peprotech (#400-01B, Peprotech, Cranbury, USA). Minocycline was from Meilun Bio (#MB1477, Meilun Bio., Dalian, Liaoning, Chian). NF-kB inhibitor PDTC was supplied from MedChem Express (#HY-18738, MCE, NJ, USA). The C3a (70-77) and rat IL-1β were dissolved in ACSF (artificial cerebrospinal fluid), while PDTC and minocycline were dissolved in saline for subsequent studies, respectively.

Antibodies

Primary antibodies for immunofluorescence studies were anti-c-Fos (1:1000, #sc-52, Santa Cruz, CA, USA), anti-PSD95 (1:100, #MAB1596, Millipore, MA, USA), anti-Synaptophysin (SYP) (1:200, #ab16659, Abcam, MA, USA), anti-CaMKIIα (1:1000, #GTX41976, GeneTex, CA, USA), anti-GAD67 (1:1000, #MAB5406, Millipore), anti-C3 (1:100, #K004838P, Solarbio, Beijing, China), anti-Iba-1 (1:100, #ab5076, Abcam), anti-CD68 (1:500, #28058-1-AP, Proteintech, Wuhan, Hubei, China). The corresponding secondary antibodies were Cy5 donkey anti-goat (1:1000, #ab97117, Abcam), 594 donkey anti–rabbit antibody (1:1000, #ab150064, Abcam), and 488 donkey anti-mouse antibody (1:1000, #ab150105, Abcam). Primary antibodies for western blotting were anti-C3 (1:1000, #K004838P, Solarbio, Beijing, China), anti-p65 (1:1000, #8242, CST, MA, USA), anti-p-p65 (1:1000, #3033, CST), anti-H3 (1:1000, #9715, CST), and anti-GAPDH (1:1000, #5174, CST). Secondary antibodies were IRDye 680RD goat anti-rabbit

IgG (1:10000, #926-68071, LI-COR, Nebraska, USA).

Open-field test (OFT)

Open-field test was thought to reflect anxiety-related behavior. The open field test was monitored in the open field box (1 m × 1 m × 40 cm) and the open field floor was divided into 25 rectangles (15 × 15 cm) by white lines. Rats were gently put in the center of the open field and allowed to explore freely for 5 min. The time spent in the central area, and the total travelled distance was videotaped and determined with ANY-maze in each 5 min period (Stoelting, Wood Dale, IL, USA). The field should be cleaned with 75 % ethanol between each rat test.

Forced swim test (FST)

Forced swimming test (FST) was thought to assess the behavioral despair. Rats were put individually into a transparent glass beaker (30 cm in diameter and 50 cm in height) which was filled with water (25 ± 2 °C) to a height of 35 cm for 5 min. The depth of water was determined to avoid the rats from escaping and keep their tails away from the bottom. The trials were performed for 5 min in normal light conditions and were recorded on videotape and then scored by the ANY-maze (Stoelting). The total duration of immobility and climbing time were recorded during the last 4 min of the 5-min trials.

Sucrose preference test (SPT)

The sucrose preference test (SPT) was used as an indicator of anhedonia. The evaluation of sucrose preference is divided into acclimatization and testing secessions. In the adaptation secession, rats were trained to drink a sucrose solution (1%) or tap water in their cage for 24 h. After the adaptation secession, rats were deprived of water and food for 24 h and then given free to access to two identical bottles with either 1% sucrose solution or tap water for 1 h. The sucrose

preference was determined as described previously [1].

Detection of the brain distribution of PHL

Rats were intragastrical administered with PHL (50 mg/Kg) and sacrificed after 15, 30, 60, or 120 min, respectively. Brain tissue samples (about 300 mg) were weighed and homogenized in 0.9% NaCl. Samples were then centrifuged at 12000 rpm for 15 min and the supernatant was collected. An aliquot of 100 μL supernatant of brain sample were added to a 1.5 mL centrifuge tube. Then, 10 μL of Reveratrol (Internal standard, 1μg/mL) and 800 μL of 80% ethyl acetate (ethyl acetate: acetonitrile = 8:2) were added and vortex mixed for 2 min. Samples were then centrifuged for 5 min at 11000 rpm, and supernatant was transferred into new vials and concentrated by nitrogen blowing at room temperature. Subsequently, samples were re-dissolved by 100 μL acetonitrile and centrifuged for 10 min at 13000 rpm. The supernatant was collected and 1 μL was injected into LC-MS/MS system (Shimadzu) and tandem AB Sciex 6500 QTRAP mass spectrometer (Applied Biosystems) for analysis. The mobile phase was composed of water (A) and methanol (B), with a flow rate of 0.3 mL per min. The gradient program was 0 to 3 min 70% B. The transitions monitored for quantification were m/z 273.0 > 167.0 for PHL and m/z 227.0 > 143.0 for Resveratrol.

Food intake

Food intake was measured by removing the chow from the wire cage lid and weighing at each interval. Given that rats mainly feed during the dark period, the feeding was begun at the onset of the dark cycle. Cumulative food intake was measured for 12 h and 24 h.

Prehensile traction test

Prehensile traction test was used to examine muscle strength and equilibrium. The

prehensile traction test was performed as described in a previous study [2] with minor modification. A 0.3 cm diameter and 55 cm long rope was placed horizontally at a height of 50 cm above a foam pad. Then, the rat's forepaws were placed on the center of the horizontal rope. The performance of rats was scored as follows: 0 points, hangs on 0 to 2 s; 1 point, hangs on 3 to 4 s; 2 points, hangs on 5 s, no third limb up to the rope; and 3 points, hangs on 5 s and brings hind limb up to the rope.

Beam balance test

Beam balance test was carried out as a previous study [3]. Briefly, a 1.2 m long and 2.3 cm wide beam was use and placed at a height of 0.5 m above the floor. The rats were placed on the beam for a maximum of 60 s and analyzing the performance with a score from 0 to 6. Rats were scored for as follows: 0 points, balances with steady posture; 1 point, grasps side of beam; 2 points, hugs the beam and one limb falls down from the beam; 3 points, hugs the beam and two limbs fall down from the beam, or spins on beam; 4 points, attempts to balance on the beam but falls off (>40 s); 5 points, attempts to balance on the beam but falls off (> 20 s); 6 points, no attempt to balance or hang on to the beam (< 20 s).

MRI image acquisition and analysis

Before scanning, every rat was put into an inhalation chamber to have a preoperative anesthesia with 5% isoflurane and positioned on the MRI scanner bed by a tooth bar. After that, a continuous 1.5% isoflurane was adopted to retain anesthesia during the MRI scanning, and the rat brain was scanned by a 7.0T MRI scanner (Bruker). High resolution structural images were obtained by using a RARE T2-weighted sequence with the following parameters: repetition time (TR) = 9000 ms, effect echo time (TEeff) = 48 ms, RARE factor = 8, average number = 8, number

of coronal slices = 54, slice thickness/gap = 0.5/0 mm, field of view (FOV) = $32 \times 32\ mm^2$, voxel size = $125 \times 125\ \mu m^2$.

The medial prefrontal cortex (mPFC) volume was calculated with an atlas-based VBM-DARTEL method, which has been demonstrated with high test-retest reliability across the rat brain in our previous study [4]. Briefly, the voxel size of T2-weighted images was enlarged 10 times to adapt to the human brain processing in SPM 12. Subsequently, T2-weighted image was registered to the SIGMA template [5] with an affine transformation, and segmented into gray matter (GM), white matter (WM) and cerebrospinal fluid (CSF) according to the tissue probability map followed a unified segmentation method. Next, the DARTEL algorithm was applied on the group-level segmented gray matter images to improve the normalization into SIGMA template, and 2 mm FWHM Gaussian kernel was used to smooth all individual images. Finally, based on the SIMGA atlas, the position of mPFC was located and its volume in every rat was calculated from the modulated and smoothed images.

Golgi Staining

Golgi Staining was conducted with FD Rapid GolgiStain™ Kit (FD Neuro Technologies, Columbia, MD, USA) according to the protocols. Briefly, one day after behavioral tests, freshly dissected brains were treated with Golgi solution (A and B) for 2-week in the dark and then transferred to solution C in the dark for 1-week. Brains were subsequently cut to 100-μm slices and incubated with Solution D and Solution E. Finally, brain sections were mounted on microscope slides. Images of Golgi stained neurons were acquired on Pannoramic SCAN II system (3DHistech, Budapest, Hungary). The dendritic spine density in the distal branches of the apical dendrites was analyzed using Image J software (NIH, Bethesda, MD, USA).

Fiber Photometry

A fiber photometry system (RWD) was applied for recording Ca^{2+} signals from mPFC neurons. After the injection of 200 nl AAV-CamKIIα-GCaMP6s virus (Brain VTA), an optical fiber was positioned immediately in a ceramic ferrule and inserted toward the mPFC (AP: 3 mm; ML: ± 0.5 mm; DV:-4 mm) through the craniotomy. The ceramic ferrule was supported with 3 ceramic screws and dental acrylic. After a period of 2-week of PHL pre-treatment followed 3-week of CMS exposure, the 1% sucrose water intake induced-changes of Ca^{2+} signals were recorded. Fluorescence signals were detected, collected and converted to $\Delta F/F$ (%) values as follows: $\Delta F/F = 100 \times (F - F_0)/F$. F_0 was the basal fluorescence signal and F was the recorded fluorescence. Data were analyzed by using MATLAB Software (MathWorks, Natick, MA, USA), and $\Delta F/F$ values were shown as heatmaps and average plots. The shaded area indicated the standard error of the mean.

Electrophysiological experiments

Rats were anesthetized and perfused with cold artificial cerebrospinal fluid (ACSF) (2.5 mM KCl, 1.25 mM NaH_2PO_4, 10 mM glucose, 26 mM $NaHCO_3$, 2 mM $CaCl_2$, and 2 mM $MgCl_2$, at pH 7.4, 310-320 mOsm). Brains were removed quickly, and cut with the vibratome VT1200S (Leica, USA) in cold artificial cerebrospinal fluid. The brain slices were incubated in ACSF at 37 °C for 45 min and then recovered for 30 min at room temperature in ACSF. After recovery, slices were put into the recordings chamber and continuously perfused with oxygenated-standard ACSF (126 mM NaCl, 2.5 mM KCl, 1.25 mM NaH_2PO_4, 25 mM glucose, 26 mM $NaHCO_3$, 2 mM $CaCl_2$, and 2 mM $MgCl_2$, at pH 7.4, 310-320 mOsm).

To record the mini postsynaptic currents (mEPSC) of layer 5 pyramidal neuron in mPFC, 1

μM tetrodotoxin (TTX) was added to oxygenated ACSF, electrodes were filled with intracellular solution (120 mM cesium methanesulfonate, 15 mM CsCl, 10 mM HEPES, 8 mM $NaCl_2$, 2 mM MgATP, 0.3 mM Na_2GTP, 10 mM TEAC, 0.2 mM EGTA, 5 mM QX-314, pH 7.25, 305-310 mOsm). The membrane potential was held at −60 mV, and the electrophysiological data were analyzed with Mini Analysis Program (Synaptosoft Inc, Fort Lee, NJ, USA).

For the LTP and LTD recordings, brain slice was placed on the center of a MED64 probe (Panasonic, Osaka, Japan) and a fine mesh anchor was used to stabilize the slice. The slice was then continually perfused with oxygenated-standard ACSF. The data acquisition software (Mobius, Alpha MED Scientific, Osaka, Japan) was adopted to monitor and maintain the noise to below 10 μV. After 15 min, a channel located in layer II/III of the mPFC was chosen as the stimulation site, and the input-output curve (I-O curve) was determined by measuring the field excitatory postsynaptic potential (fEPSP) amplitude or slope in response to a series of stimulation intensities starting at 10 μA. The stimulation pulse (duration of 0.2 ms, 0.033 Hz) chosen for the baseline measurements was adjusted to produce 30% of the maximal amplitude. After the baseline responses stabilized for 10 minutes, LTP was induced by theta-burst stimulation, including 10 trains of burst with 4 pulses at 100 Hz (200 ms intervals), whereas LTD was induced by low frequency stimulation (LFS), including a series of 900 pulses at a frequency of 1 Hz. The activities of LTP and LTD were recorded for at least 60 minutes, respectively.

Sholl analysis

Sholl Analysis was carried out as described previously [6]. In short, the 40 μm-thick brain sections from the mPFC were immunostained with Iba-1 antibody and then conducted for confocal imaging. Consecutive confocal z-stacks of Iba-1 positive cells were obtained with a TCS

SP8 confocal microscope (Leica Microsystems, Wetzlar, Germany), and the maximum intensity projections of individual cells were converted to the detailed images of cells with all their branches by Image J software (NIH). The center of the soma was regarded as the center of all concentric circles. The number of intersections was calculated from 5 μm of soma center and subsequent circles set at 5 μm. Using the Sholl analysis plugin [7], the number of total branch points and the number of intersections of cell proccesses with consecutive concentric circles per microglia were recorded. Three microglia were selected from each rat, six rats per group. The average value per rat was used for statistical analysis.

Imaging analysis

To quantify the number of positive cells, the co-localization of c-fos and NeuN, c-fos and CamkIIα as well as c-fos and GAD67, the same level of brain slices were selected and imaged. Images were acquired by a confocal microscope (Leica, USA) with 0.2 μm z-steps, and consecutive z-stack pictures (8 stacks) were used to create a maximum intensity projection image with Leica confocal software. The number of positive cell and co-localization were counted in a double-blind manner and subsequent quantified for three to five rats in each group. The average value per rat was used for statistical analysis.

To determine the co-localization of PSD95 and SYP, C3 and PSD95, the same level of brain slices were imaged on a Nikon Structure Illumination Microscopy (SIM) (Nikon, Tokyo, Japan) equipped with a 100 × oil-immersion lens (numerical aperture 1.49) and laser beams (405, 488, 561, and 640 nm) to obtain 3D images. Image stacks (about 675 pictures) of 6 μm height with 9 images per plane (3 phases, 3 angles) and a z-step of 0.24 μm were acquired. Then, images were analyzed with IMARIS software (Bitplane, Zurich, Switzerland) and the "Spots" function of

IMARIS was used to 3D reconstruct the PSD95, SYP, and C3 positive puncta. MATLAB was used to determine the number of co-localized (≤ 200 nm distance between spot centers of two channels) spots. Finally, we used Nikon's N-SIM microscopy system "Reconstruct Slice" function to convert 2D images. Two images per rat were reconstructed, five rats per group. The average value per rat was used for statistical analysis.

Microglial engulfment was determined by using IMARIS software (Bitplane) to create 3D reconstruction of microglia and CD68 as described in a previous study [8]. We first acquired image stacks (about 900 pictures) of 6 μm height with 9 images per plane (3 phases, 3 angles) on a Nikon Structure Illumination Microscopy (SIM) (Nikon) from the same level of brain slices. Then, IMARIS software was used to create a 3D surface rendering of the microglia and CD68. Next, we used MATLAB plugin "Surface-Surface Contact Area" to 3D reconstruct CD68 occupancy in microglia. The $PSD95^+$ puncta were reconstructed using the IMARIS "Spots" function, and MATLAB plugin "Distance between Spots And Surface" was used to assess the number of $PSD95^+$ puncta entirely within CD68 occupancy in microglia surface. Two images per rat were reconstructed, five rats per group. The average value per rat was used for statistical analysis.

RNA sequencing

Total RNA was prepared from mPFC tissue with TRIzol Reagent (Invitrogen, Carlsbad, CA, USA) according to the manufacturer's protocols. RNA integrity was evaluated using the RNA Nano 6000 Assay Kit of the Bioanalyzer 2100 system (Agilent Technologies, CA, USA). The RNA-seq transcriptome library was constructed by using the NEBNext® Ultra™ RNA Library Prep Kit for Illumina® (New England Biolabs) with 1 μg of total RNA. RNA sequencing was

performed using a NovaSeq6000 and High Output v2 kit (Illumina). All RNA-seq raw data were uploaded in the Gene Expression Omnibus under accession number (GSE193284).

The clean data were acquired by removing raw reads including adapter, reads containing ploy-N and low quality reads from raw data through in-house perl scripts. To determine the differentially expressed genes (DEGs) between two different samples, the expression of each gene transcript was determined according to the fragments per kilobase million (FRKM) mapped reads method. DESeq R package (1.10.1) was adopted to identify DEGs in all the transcriptomic data. The *P* values were adjusted with the Benjamini & Hochberg method. The corrected *P*-value of 0.05 and absolute fold-change of 2 were set as the threshold for significantly differential expression.

Real-time PCR analysis

Total RNA was prepared with the TRIzol reagent (Invitrogen). Then, 1 μg of total RNA was reverse transcribed into cDNA with a first-strand cDNA synthesis kit (Roche, Basel, Switzerland). The relative level of mRNAs was determined by the SYBR Green Master Mix (Life Technologies). The relative expression of genes of interest was calculated by $2^{-\Delta\Delta CT}$ method and GAPDH gene was chosen as the housekeeping gene. The primer sequences used in real-time PCR were provided in Table S1.

Western blot analysis

Brain tissues or cultured cells were lysed in RIPA lysis buffer (Sigma-Aldrich) containing a cocktail of protease (Roche) and phosphatase (Sigma-Aldrich) inhibitors. The protein concentration of the samples was determined with a BCA Protein Assay Kit (Beyotime, Shanghai, China). The linear range for each tested protein was performed as described in a previous study

with minor revision [9]. Briefly, different amounts of total protein (10, 20, 30, and 40 μg) were separated on an SDS-PAGE gel and transferred to polyvinylidene fluoride (PVDF) membrane. The membranes were blocked with 5% nonfat milk or BSA in Tris-buffered saline (TBS) containing 1% Tween (T-BST) for 2 h at room temperature, the membranes were then incubated with primary antibodies, including C3, SYP, PSD95, p65, p-p65 and GAPDH overnight at 4 °C, respectively. The membrane was washed with TBST for 3 times, and then incubated with secondary antibody (LI-COR) for 1 h at room temperature. After washing for 3 times, the signals were examined with an Odyssey Infrared Imaging system (LI-COR), and the tested protein band densities were analyzed by Image J software (NIH). The linear range for each tested protein was calculated by analyzing tested protein band densities and the corresponding total protein amounts. After determining the linear range of protein, corresponding protein amounts were selected to perform western blot analysis. Finally, the intensity of tested protein signal was normalized to the loading control.

Enzyme-linked immunosorbent assay (ELISA) and MDA (Malondialdehyde) measurement

The mPFC brain tissues were homogenized in ice-cold PBS, and the homogenate was centrifuged at 12000 × g for 15 min at 4 °C. The supernatant was then collected and used to detect the level of cytokine IL-1β, TNF-α and MDA. For the level of IL-1β and TNF-α, the IL-1β and TNF-α ELISA kit (Proteintech, Wuhan, Hubei, China) were used according to the instructions. Then, the absorbance at 450 nm was detected on a microplate reader (Bio-Tek Instruments, Winooski, VT, USA). For the level of MDA, the Lipid Peroxidation MDA assay kit (Beyotime) was used and the absorbance at 535 nm was determined on a microplate reader (Bio-Tek Instruments).

Cell culture

BV2 murine microglial cells were maintained in Dulbecco's modified Eagle's medium (DMEM)/F12 medium (Corning, Manassas, VA, USA) supplemented with 10% fetal bovine serum (FBS) (Life Technologies) and 1% penicillin-streptomycin solution (Life Technologies). The cells were maintained in a humidified incubator at 37 °C with 95% air and 5% CO_2.

Rat primary microglia were obtained from 1-day-old SD rats. Briefly, rats were anesthetized and their brains were quickly removed. The brain tissues were dissociated by gentle trituration using a pipette. Then, cell suspension was filtered through a 40-μm cell strainer. The isolated cells were transferred to a 75-cm^2 flask with DMEM/F-12 medium (Corning) containing 10% FBS and 1% penicillin and streptomycin in a humidified incubator. After 14-day of culture, primary microglia were collected by shaking the flask for 2 h.

Primary astrocytes were prepared from postnatal 1-day-old SD rats. Briefly, rats were anesthetized and their brains were quickly removed. The rat brain were dissociated by scissors and then incubated with 0.25% trypsin-EDTA at 37 °C for 5 min. The isolated cells were resuspended in DMEM/F12 medium (Corning) supplemented with 10% FBS and 1% penicillin/streptomycin in a humidified incubator. After 14-day of culture, cultures reached confluence and astrocytes were obtained by shaking the flask for 2 h to dislodge the microglia and oligodendrocytes.

Isolation of cytosolic and nuclear fractions

Primary astrocytes and microglia were pre-treated with or without 20 μM of PHL for 24 h, and then treated with or without 50 ng/ml of IL-1β for 24 h. Then, cells were collected and cytosolic and nuclear were prepared with a NE-PER nuclear protein extraction kit (Thermo Scientific, Rockford IL, USA) according to the protocols. The protein content of the samples was

determined by a BCA Protein Assay Kit (Beyotime). Next, western blot was carried out to detect the level of target proteins in nucleus and cytoplasm.

Chromatin immunoprecipitation (ChIP) assay

ChIP experiments were performed according to the protocols provided with EZ-Magna ChIP™ A/G Chromatin Immunoprecipitation Kit (Millipore). Briefly, 1×10^7 cells were cross-linked by using 1 % formaldehyde for 10 min, and the crosslinking reaction was stopped by 0.125 M glycine for 5 min. Then, cells were lysed and sonicated into DNA fragments of 200 to 1,000bp. After centrifugation, the supernatant was harvested and incubated with normal IgG serum (1:100, #2729, CST) or anti-p65 (1:100, #8242, CST). The DNA-protein-IgG complexes were precipitated with protein A/G magnetic beads and eluted with elution buffer. The eluents were then reverse-crosslinked and DNA was purified. Finally, real-time PCR was carried out with DNA extracted from the immunoprecipitated chromatin. The relative amount of immunoprecipitated DNA was analyzed by normalization to input. The primer sequences used in this section to detect the NF2 site were as follows: 5'-GTTACTGGGACCCAGCTAATTGT-3', 5'-GGGTGAGTAACCCCAGGCACAT -3'.

Stereotaxic surgery and drug treatment

Rats were anesthetized and head-fixed in a stereotactic apparatus (RWD). For cannula implantation, the rat skull was exposed and a small hole was drilled with a dental drill around the region of interest. According to the atlas of Paxinos and Franklin, the stainless steel guide cannulas (RWD) were bilaterally implanted into the mPFC region and fixed to the skull with dental cement and anchor screws. Stainless steel obturators were inserted into the guide cannulas until an injection was to be performed. When the intracranial injection was started, the obturators

were removed and the injection cannulas were then inserted. C3a (70-77) (40 ng/μl, dissolved in ACSF), IL-1β (100 ng/μl, dissolved in ACSF) was bilaterally injected into mPFC (0.2 μl per side) at the speed of 0.1 μl/min by a microsyring once every two days, respectively. After injection, the cannulas were left for another 5 min to allow drug diffusion. PDTC was intraperitoneal injected into rats at the dose of 100 mg/kg. As to minocycline treatment, rats were intraperitoneally injected with saline and minocycline (50 mg/kg, diluted in saline) once daily.

Supplementary Tables

Table S1

Primers used for real-time PCR

Gene name	Primer sequence (5′-3′)	Orientation
C3	ATCGAGGATGGTTCAGGGGA	Forward
	GCTTGTGGAGGTAGGACTTG	Reverse
C1qa	TCGGGACAGCAACCAAAC	Forward
	TGCAGATGATTGAGAACCTTGG	Reverse
C1qb	CTTTCTGCTTTTCCCTGACATG	Forward
	AGTCAAAGGGCAGTGTCTG	Reverse
Cx3cr1	GTCTTCACGTTCGGTCTGGT	Forward
	ACAAAGAGCAGGTCGCTCAA	Reverse
Cfh	CAGACTGTTGCCACAAAGCA	Forward
	AGTGTGTCTTCCACTCACTGT	Reverse
GAPDH	GACCACCCAGCCCAGCAAGG	Forward
	TCCCCAGGCCCCTCCTGTTG	Reverse

Table S2

Primers used to construct reporter constructs

Reporter gene name	Primer sequence (5′-3′)	Orientation
C3-Luc	ACGGGGTACCAACCCGCCCCTTCCATGCAGTTC	Forward
	ACCCAAGCTTGGTGGGCAGTGGTAAGCCAGGGCT	Reverse
Mut1-Luc	ATTGAGGAATAGACTGACAGAAGTGCTGGGTTACTGGGA	Forward
	TCTGTCAGTCTATTCCTCAATACCCCCCAGGTCTTGCTAA	Reverse
Mut2-Luc	ATTACCAGATTAGGTTCAGCATCTGGCCTTGACCTCGA	Forward
	GCTGAACCTAATCTGGTAATTGTTGTACTTGGTTGCAC	Reverse
Mut3-Luc	ATTGCAGAATCTGGGGCAGGAACTGGGAAGGGCCAGAGA	Forward
	CCTGCCCCAGATTCTGCAATAGATTTCGCAACGTGGTTTC	Reverse
Mut4-Luc	ATTAAGGGATAGAGAGGAGAACCACATAAAAAGGCAGTGGC	Forward
	TCTCCTCTCTATCCCTTAATAGTTCCTGCCCCAGGGGCTGC	Reverse

Restriction sites are underlined.

Table S3

Effects of CMS and PHL on the rat motor performance in beam balance and prehensile traction tests

Group	Number	Beam balance scores	Prehensile traction scores
Veh	8	0.50±0.27	2.25±0.23
Veh + CMS	8	0.75±0.25[1]	2.13±0.21[1]
PHL + CMS	8	0.63±0.18[2, 4]	2.38±0.25[2, 4]
PHL	8	0.63±0.26[3]	2.25±0.34[3]

Values are presented as mean ± SEM. Statistical Analysis was performed by One-way ANOVA followed by the Turkey's method for post hoc multiple comparison tests. [1] No significant difference *vs.* Veh group. [2] No significant difference *vs.* Veh + CMS group. [3] No significant difference *vs.* Veh group. [4] No significant difference *vs.* Veh group.

Table S4

Effects of CMS and PHL on the food intake at 12 h and 24 h

Group	Number	12 h	24 h
Veh	8	20.91±1.14	29.76±1.46
Veh + CMS	8	14.18±0.43****	24.18±0.63****
PHL + CMS	8	17.98±0.54*	27.84±0.82*
PHL	8	21.26±0.91^{ns}	29.18±0.69^{ns}

Values are presented as mean ± SEM. Statistical Analysis was performed by One-way ANOVA followed by the Turkey's method for post hoc multiple comparison tests. **** Significant difference *vs.* Veh group. * Significant difference *vs.* Veh + CMS group. ^{ns} No significantly difference *vs.* Veh group.

Supplementary Figures and Legends

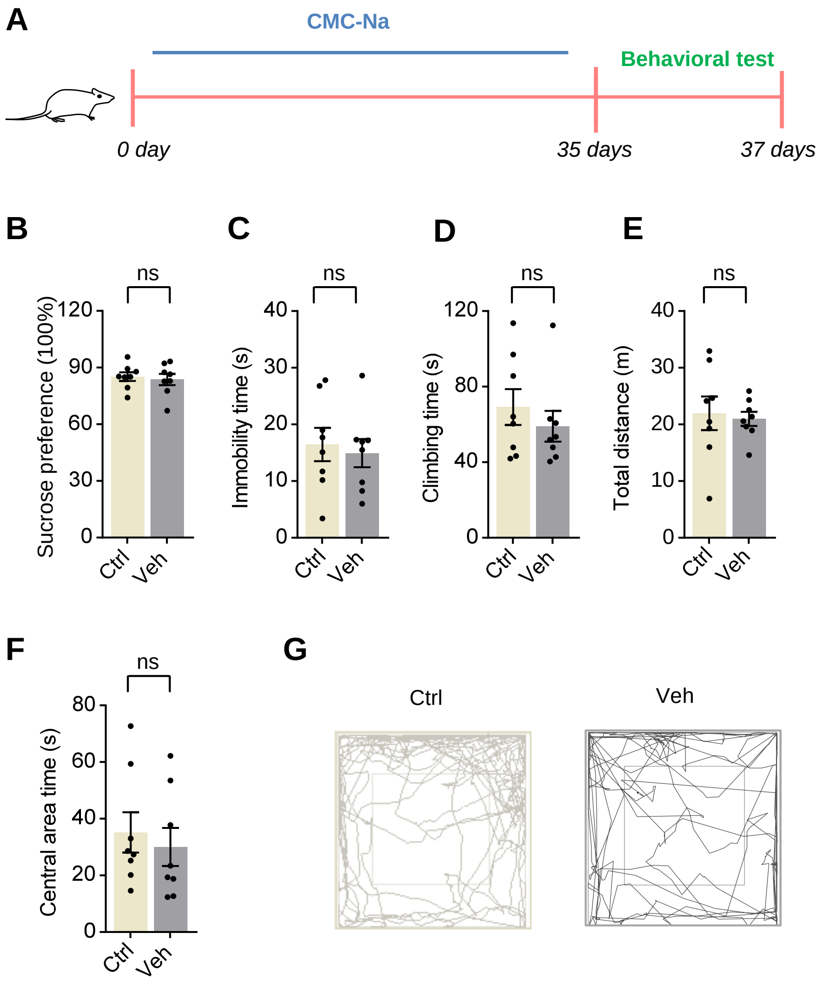

Figure S1. CMC-Na does not influence the normal rat behaviors in the SPT, FST and OFT. (**A**) Schematic procedure for investigating the effects of 0.5 % CMC-Na on the behaviors of normal rats. (**B**) Sucrose preference was measured in normal rats after CMC-Na treatment (n = 8 rats per group). (**C-D**) The FST was evaluated in normal rats after CMC-Na treatment (n=8 rats per group). The immobility time spent in the FST (**C**). The climbing time spent in the FST (**D**). (**E-F**) The OFT was performed in normal rats after CMC-Na treatment (n = 8 rats per group). The total distance traveled in the open field area (**E**). Time spent in the center (**F**). (**G**) Representative animal traces of rat movement in the OFT. Data are expressed as mean ± SEM. Two-tailed unpaired t test (**B-F**). ns, no significant difference.

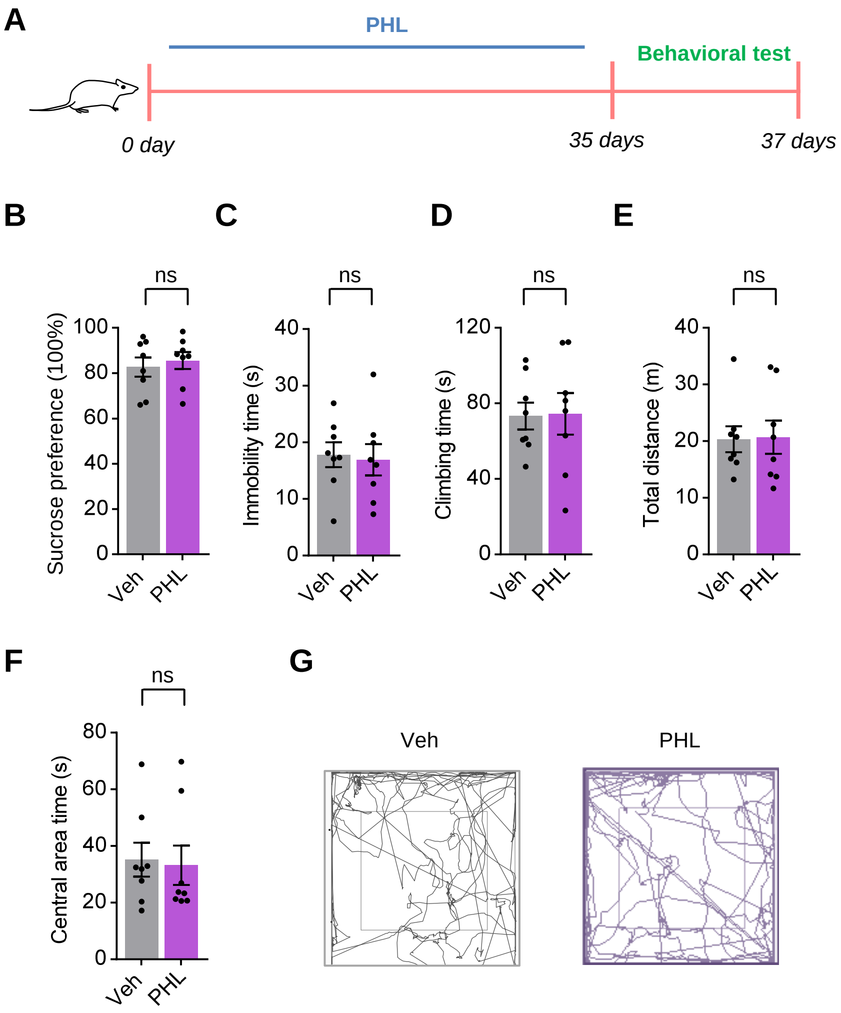

Figure S2. PHL does not influence the rat behaviors in the SPT, FST and OFT. (**A**) Schematic procedure for investigating the effects of 20 mg/kg PHL on the rat behaviors. (**B**) Sucrose preference was measured after PHL treatment (n = 8 rats per group). (**C**-D) The FST was evaluated after PHL treatment (n = 8 rats per group). The immobility time spent in the FST (**C**). The climbing time spent in the FST (**D**). (**E-F**) The OFT was performed after PHL treatment (n = 8 rats per group). The total distance traveled in the open field area (**E**). Time spent in the center (**F**). (**G**) Representative animal traces of rat movement in the OFT. Data are expressed as mean ± SEM. Two-tailed unpaired t test (**B-F**). ns, no significant difference.

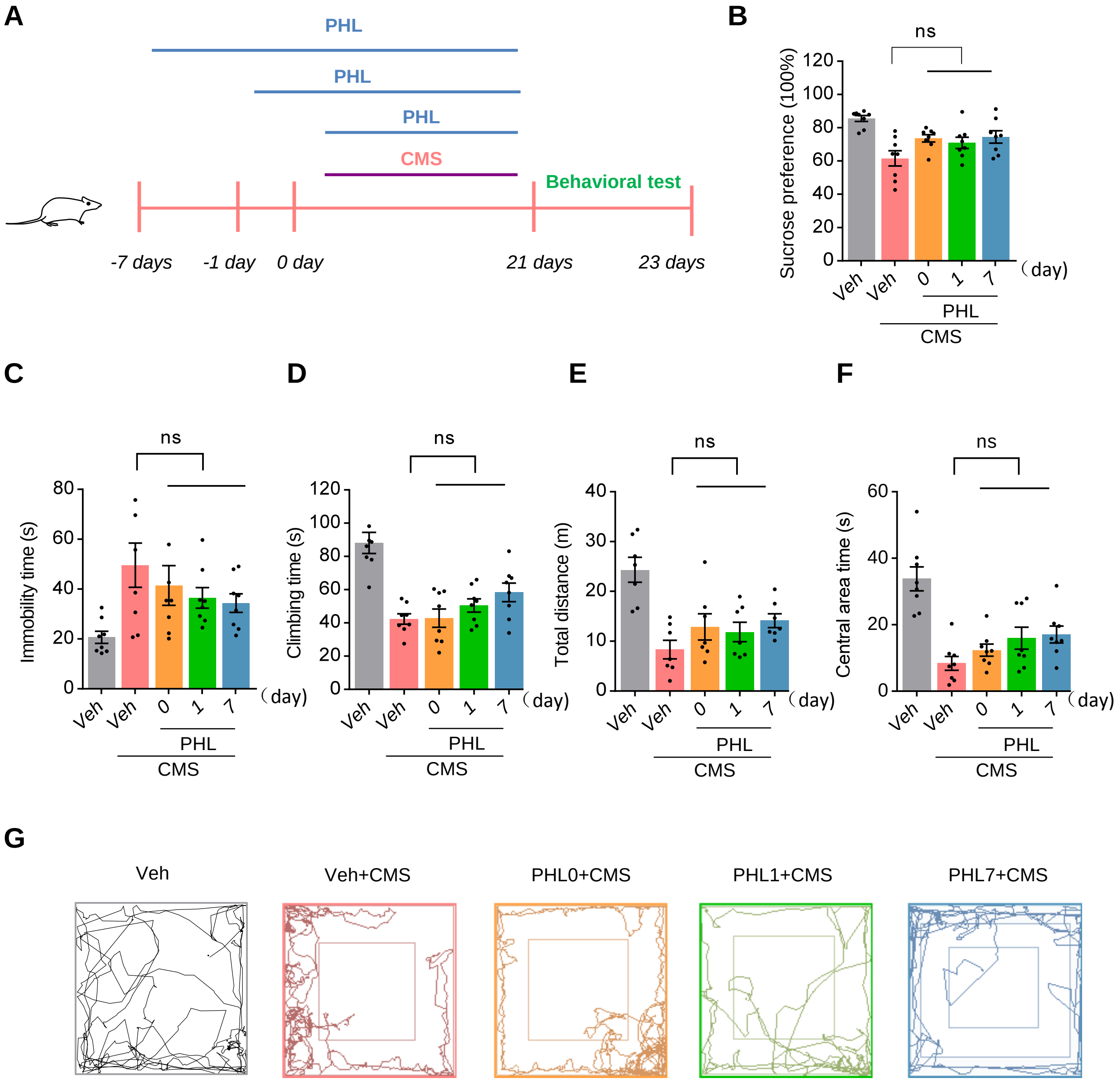

Figure S3. The pre-treatment time of PHL influences the protective effects of PHL on the CMS-induced depression-like behaviors. (**A**) Schematic procedure for investigating the pre-treatment time of PHL (20 mg/kg) on the CMS-induced depression-like behaviors. (**B**) Sucrose preference was measured in CMS rats after the indicated times of PHL pre-treatment (n = 8 rats per group). (**C-D**) The FST was evaluated in CMS rats after the indicated times of PHL pre-treatment (n = 8 rats per group). The immobility time spent in the FST (**C**). The climbing time spent in the FST (**D**). (**E-F**) The OFT was performed in CMS rats after PHL pre-treatment (n = 7 to 8 rats per group). The total distance traveled in the open field area (**E**). Time spent in the center (**F**). (**G**) Representative animal traces of rat movement in the OFT. Data are expressed as mean ± SEM. One-way ANOVA with the Tukey's post hoc test (**B-F**). ns, no significant difference.

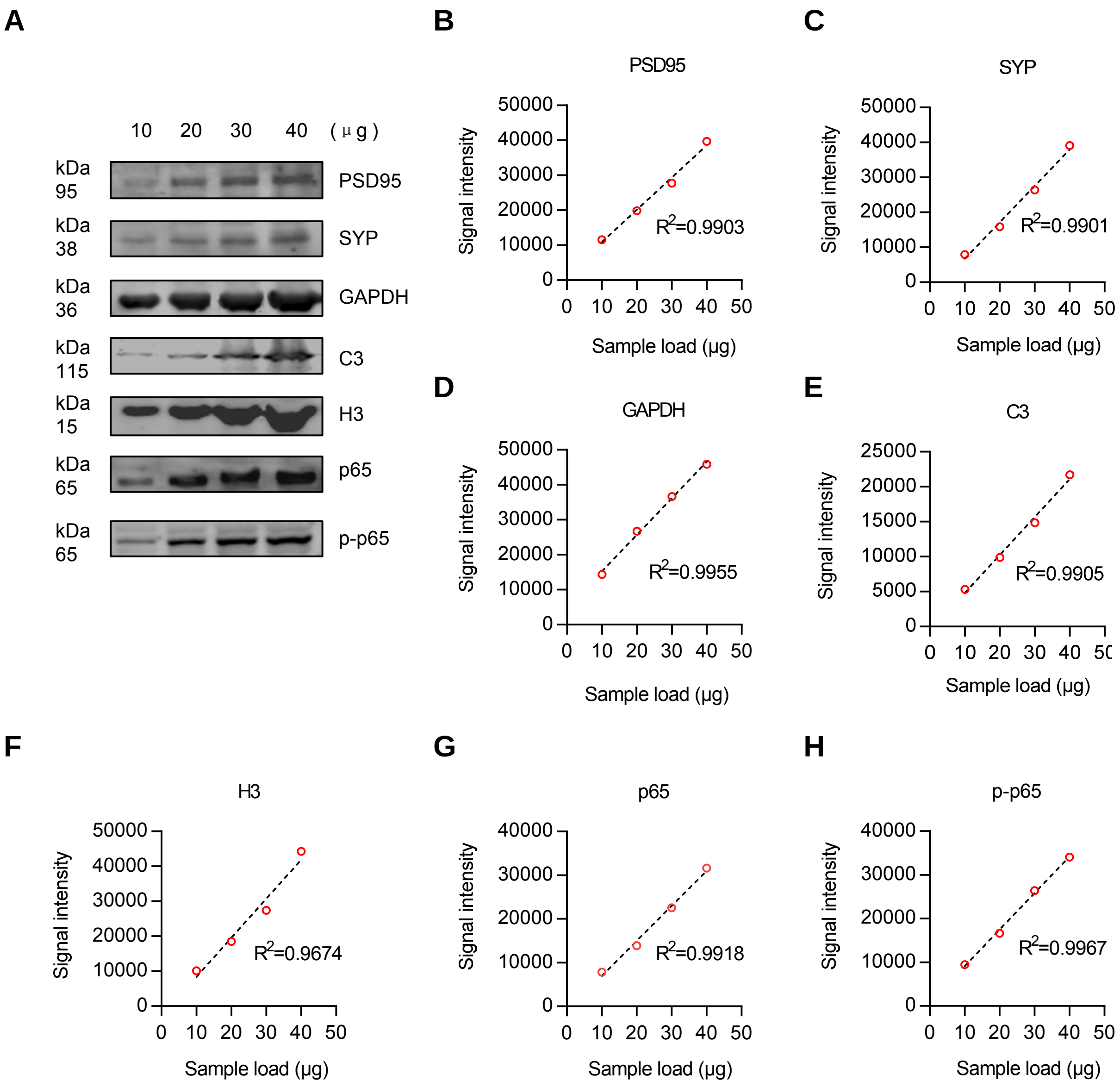

Figure S4. Determination of the linear range for each target protein in western blot analysis. (**A**) Western blotting of rat mPFC tissue total protein (10 to 40 μg) by using anti-PSD95, anti-SYP, anti-GAPDH, anti-C3, anti-H3, anti-p65, and anti-p-p65 antibodies. (**B**-**H**) R-squared values were used to evaluate the linear regression for each of target protein analyzed in (**A**). The signal intensity value was from the mean value of 3 repeats.

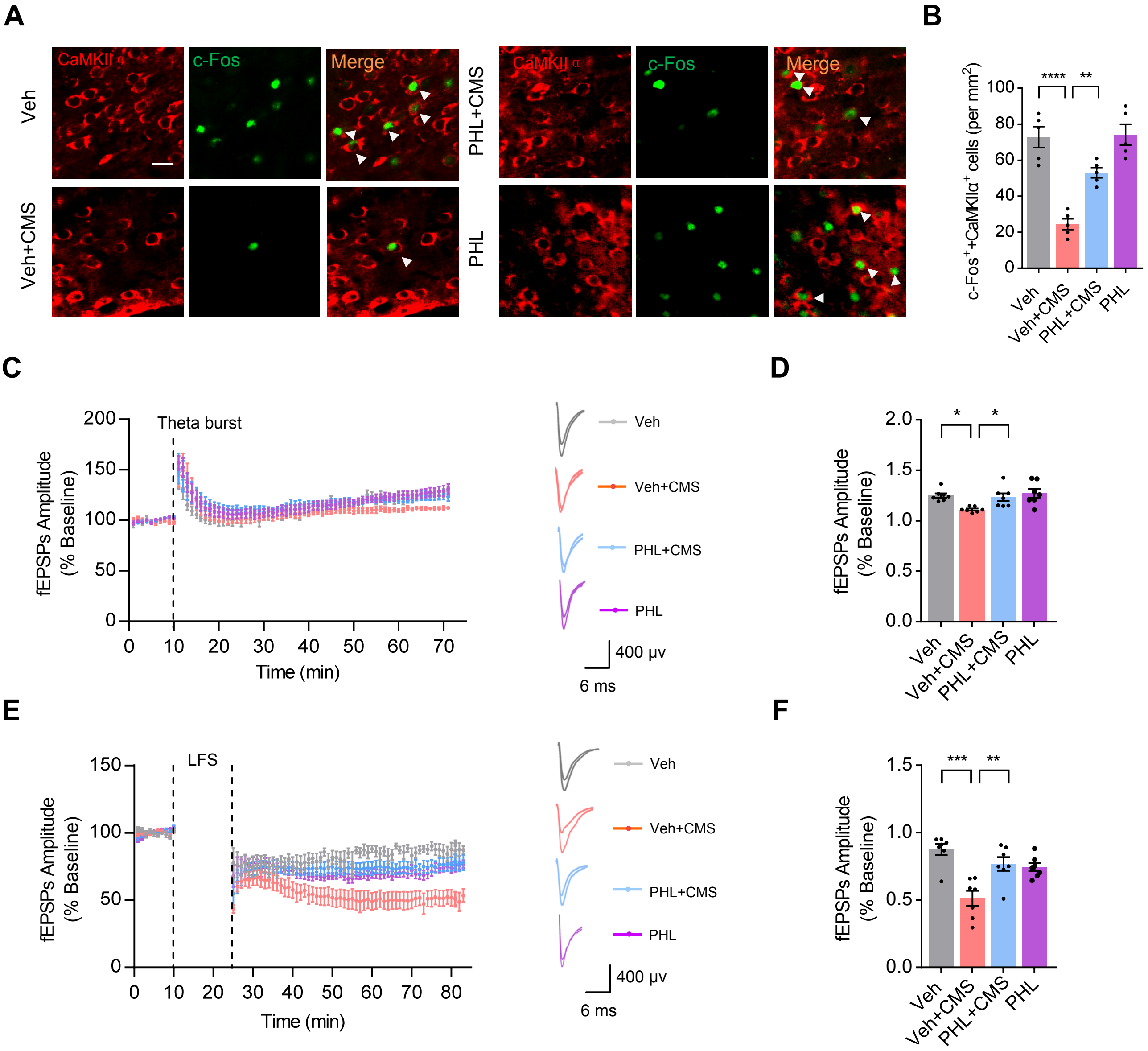

Figure S5. PHL improves the neuronal activity in mPFC after CMS exposure. (**A-B**) Representative images (**A**) and quantification (**B**) of CaMKII α and c-Fos double-positive cells in the mPFC of different group rats (n = 3 rats per group). Scale bar =20 μm. (**C, E**) The amplitude of fEPSPs recorded in the mPFC of different group rats after the induction of LTP (**C**) and LTD (**E**) (n = 7 slices from 3 to 4 rats per group). (**D, F**) The mean amplitude of fEPSPs during the last 10 min after the induction of LTP (**D**) and LTD (**F**) in different group rats (n = 7 slices from 3 to 4 rats per group). Data are shown as mean ± SEM. $^{*}P < 0.05$, $^{**}p < 0.01$, $^{***}p < 0.001$, $^{****}p < 0.0001$.

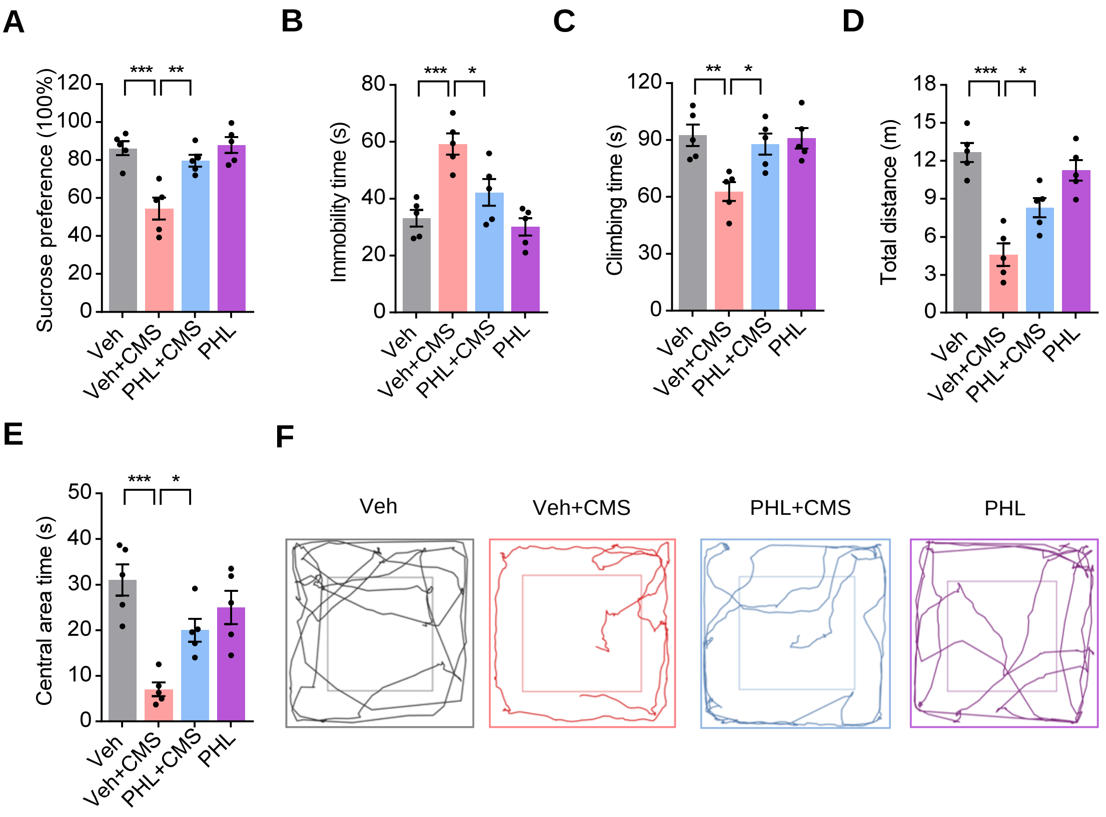

Figure S6. The CMS exposure leads to depression-like behaviors after the injection of AAV-CamKIIα-GCaMP6s virus in the rat mPFC. (**A**) Sucrose preference was measured in different groups after the injection of virus (n = 5 rats per group). (**B-C**) The FST was evaluated in different groups after the injection of virus (n = 5 rats per group). The immobility time spent in the FST (**B**). The climbing time spent in the FST (**C**). (**D-E**) The OFT was performed in different groups after the injection of virus (n = 5 rats per group). The total distance traveled in the open field area (**D**). Time spent in the center (**E**). (**F**) Representative animal traces of rat movement in the OFT. Data are expressed as mean ± SEM. One-way ANOVA with the Tukey's post hoc test (**A-E**). $^{*}p < 0.05$, $^{**}p < 0.01$, $^{***}p < 0.001$.

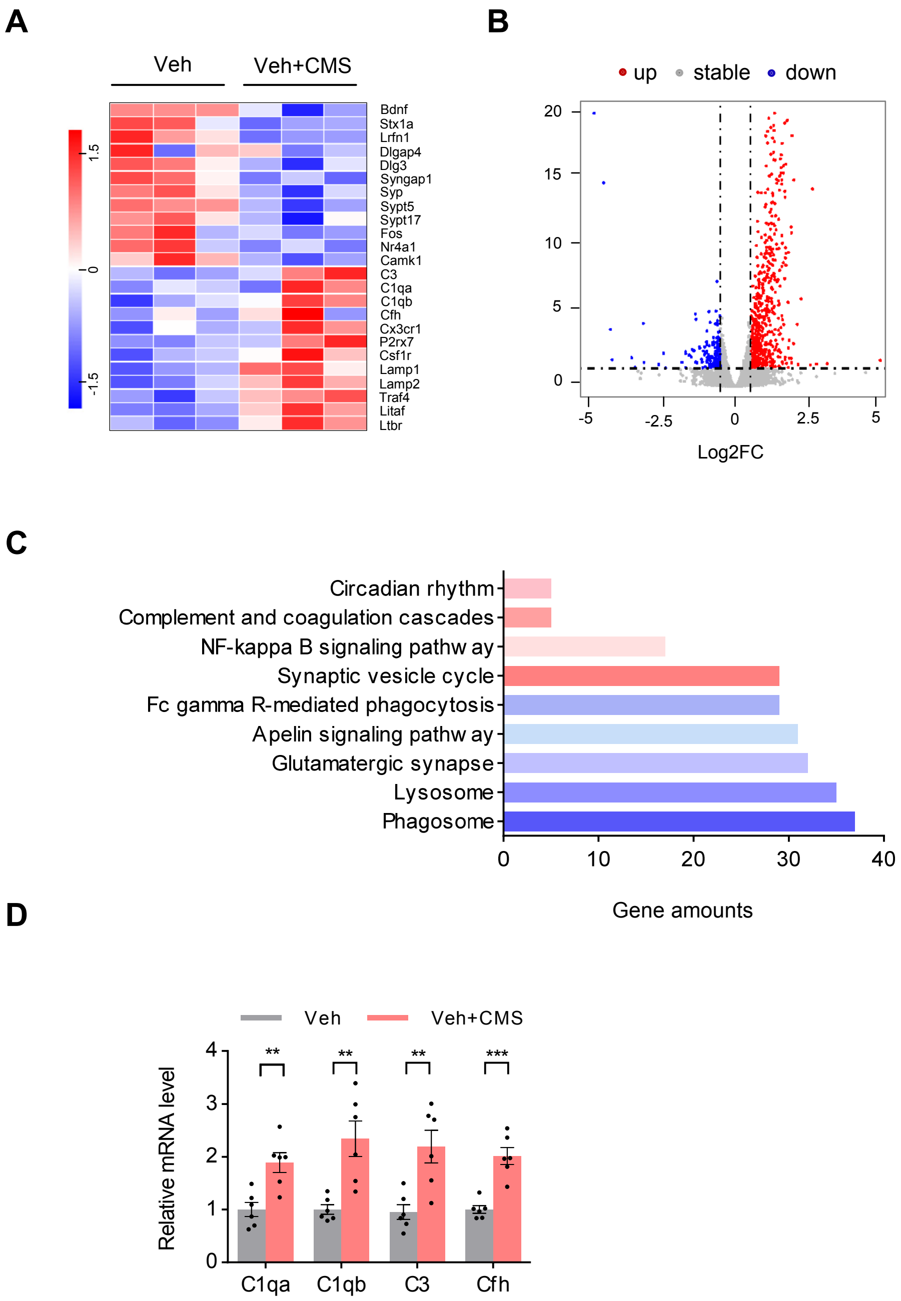

Figure S7. CMS exposure changes the gene expression profile in the rat mPFC tissues. (**A**) Heat maps of the differentially expressed genes determined by RNA-seq from the mPFC tissues after CMS exposure. Each group contains 3 rats. (**B**) Volcano plot showed the significantly differentiated genes. Up-regulated genes were shown in red, while down-regulated genes were shown in blue. (**C**) KEGG pathway analysis for DEGs in the mPFC tissues after CMS exposure. (**D**) Real-time PCR was carried out to verify several complement related genes indicated in (**A**) after CMS exposure (n = 6 rats per group). Data are expressed as mean ± SEM. $^{**}p < 0.01$, $^{***}p < 0.001$.

A

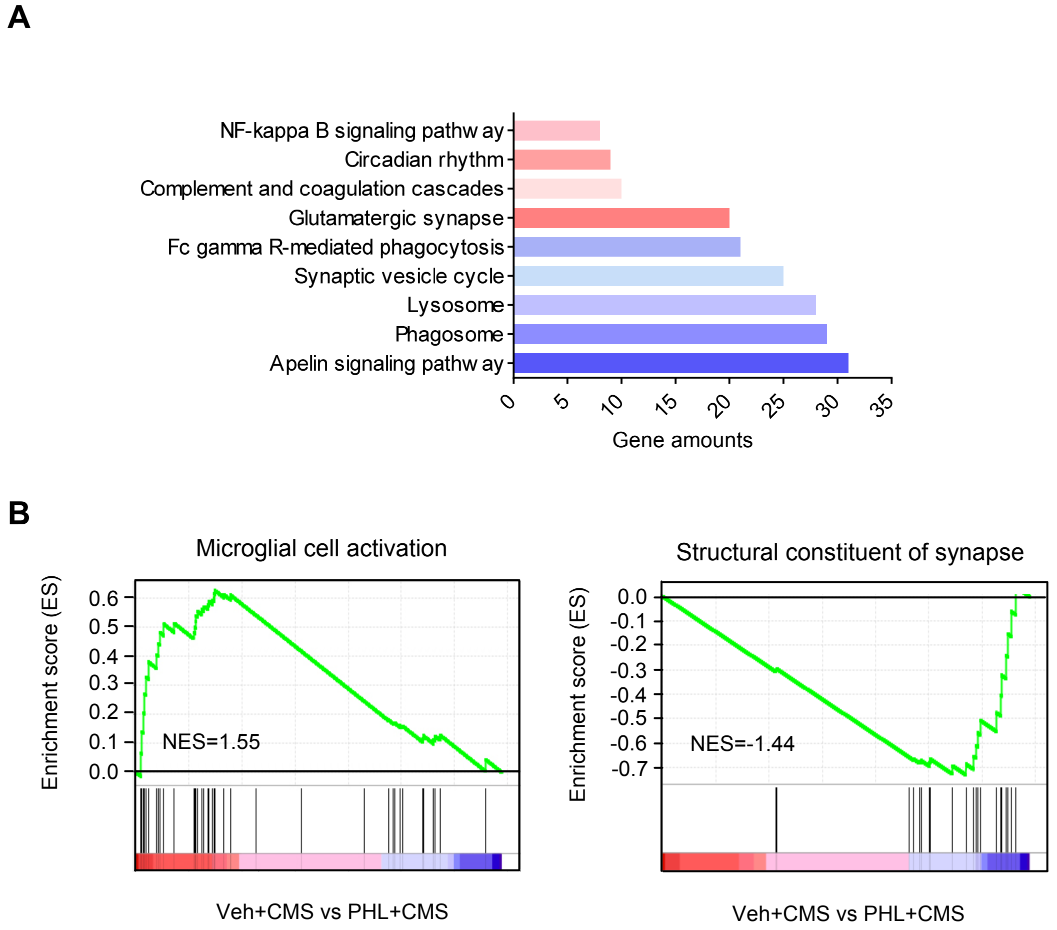

Figure S8. KEGG and GSEA analysis of RNA sequencing data. (**A**) KEGG pathway analysis for DEGs in the mPFC tissues of CMS rats after the treatment of PHL. (**B**) GSEA analyses showed that the processes of microglial cell activation and the structural constituent of synapse were changed in the mPFC tissues of CMS rats after the treatment of PHL.

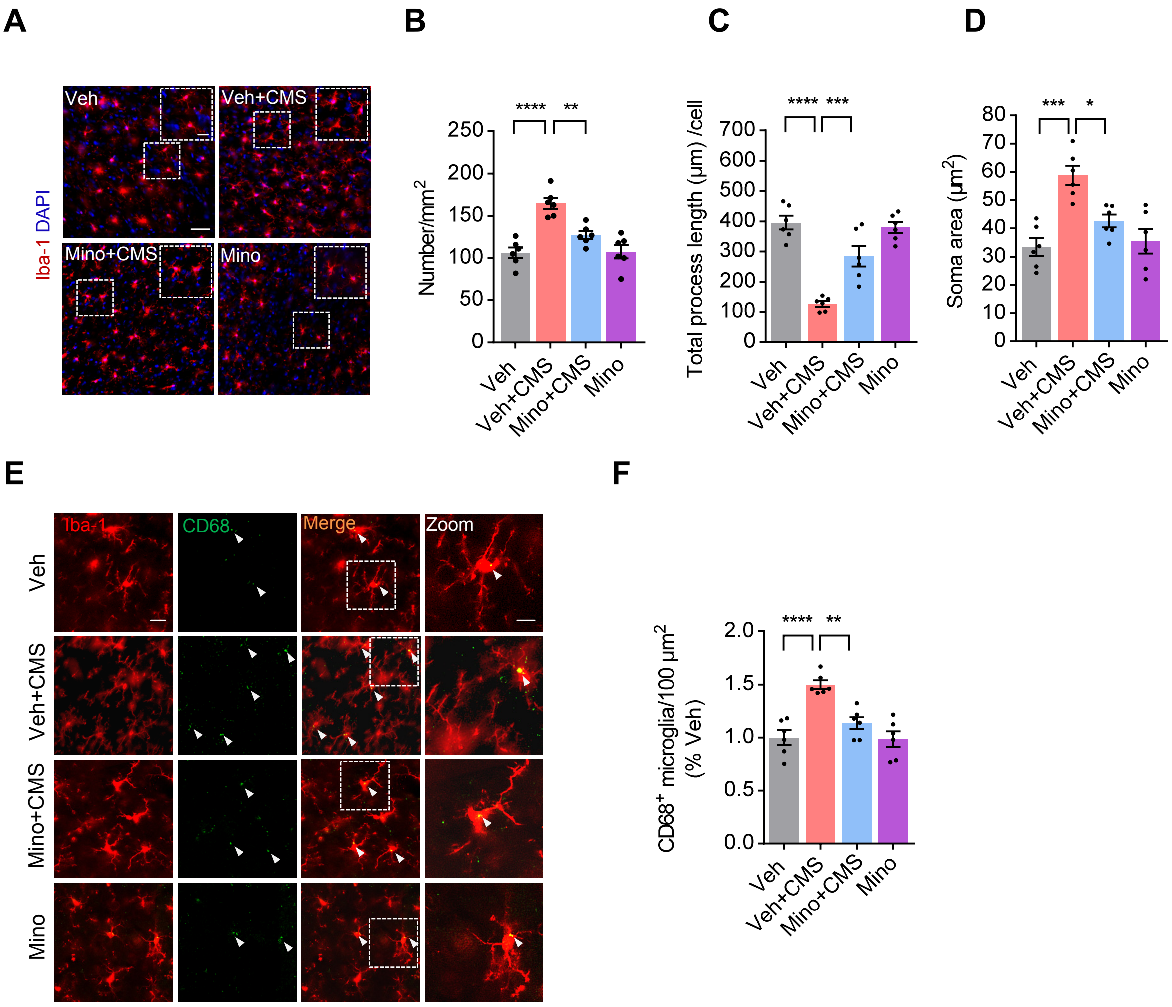

Figure S9. Minocycline represses microglia activation and phagocytosis in the mPFC of CMS rats . (**A**) Representative immunostaining images of Iba-1$^+$ positive microglia (Red) in the mPFC. Scale bar = 50 μ m. Zoom in images (bar = 20 μ m). (**B-D**) The histogram represents the quantification of number (**B**), process length (**C**), and soma area (**D**) of Iba-1$^+$ cells in the mPFC (n = 6 rats per group) . (**E**) Representative immunostaining images of Iba-1 (red) and CD68 (green) in the mPFC of different groups. Scale bar = 20 μ m. Zoom in images (bar = 10 μ m). (**F**) The CD68$^+$ microglia were presented in different groups (n = 6 rats per group). Data are expressed as mean ± SEM. One-way ANOVA with the Tukey's post hoc test (**B**-**D**, **F**). $^*p < 0.05$, $^{**}p < 0.01$, $^{***}p < 0.001$, $^{****}p < 0.0001$.

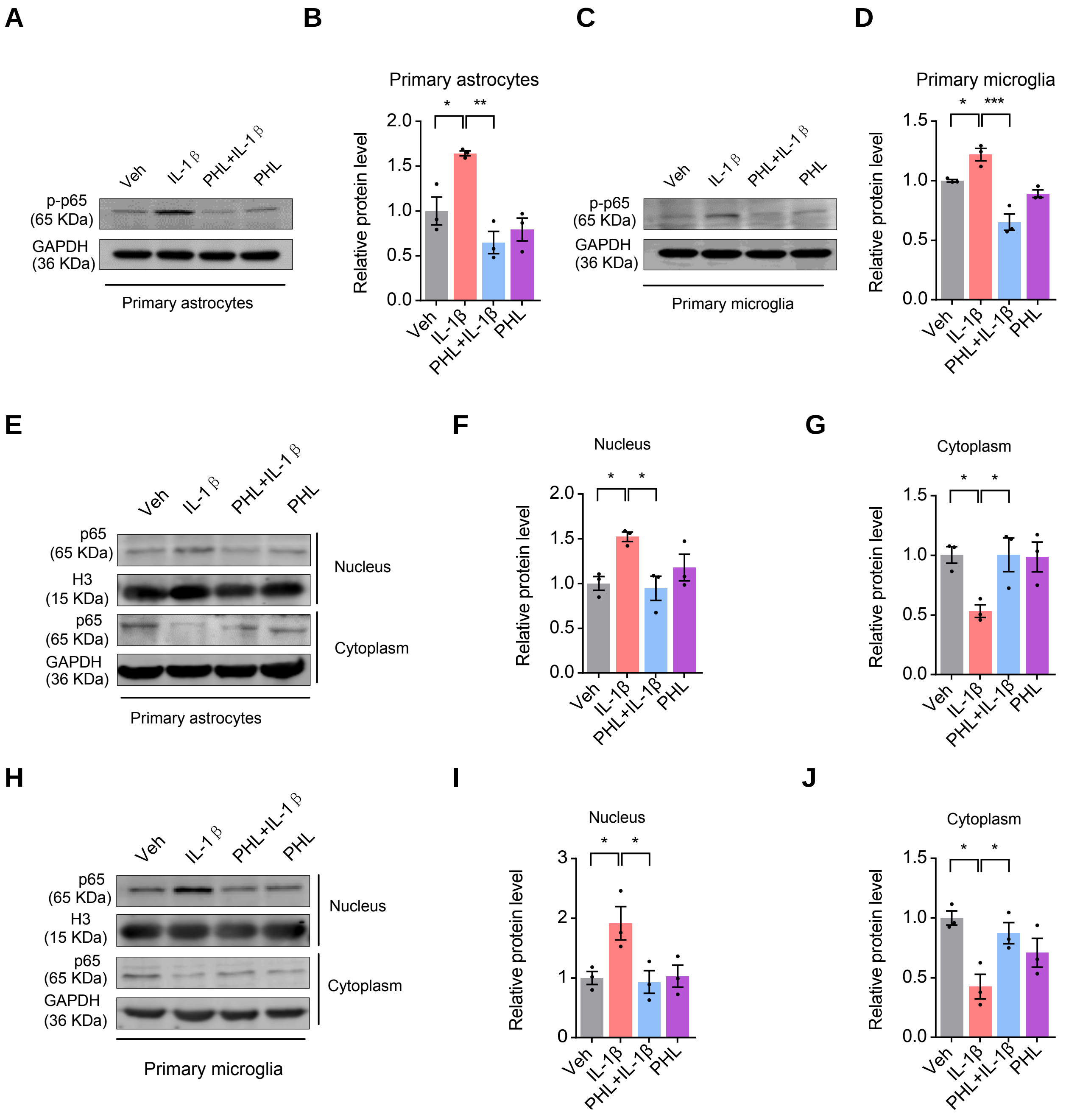

Figure S10. PHL reduces the IL-1β-induced phosphorylation of p65 and the translocation of p65 to nuclear in primary microglia and astrocytes. (**A**, **C**) The level of p-p65 was detected by western blot in primary astrocytes (**A**) and microglia (**C**). (**B**, **D**) Relative quantitative analysis of p-p65 protein level in primary astrocytes (**B**) and microglia (**D**). (**E**) The level of p65 was detected by western blot in the nucleus and cytoplasm of primary astrocytes. (**F-G**) Relative quantitative analysis of p65 protein level in the nucleus (**F**) and cytoplasm (**G**) of primary astrocytes. (**H**) The level of p65 was detected by western blot in the nucleus and cytoplasm of primary microglia. (**I-J**) Relative quantitative analysis of p65 protein level in the nucleus (**I**) and cytoplasm (**J**) of primary microglia. Data are shown as mean ± SEM, n = 3 per group. One-way ANOVA with the Tukey's post hoc test (**B**, **D**, **F**, **G**, **I**, **J**). $^{*}p < 0.05$, $^{**}p < 0.01$, $^{***}p < 0.001$.

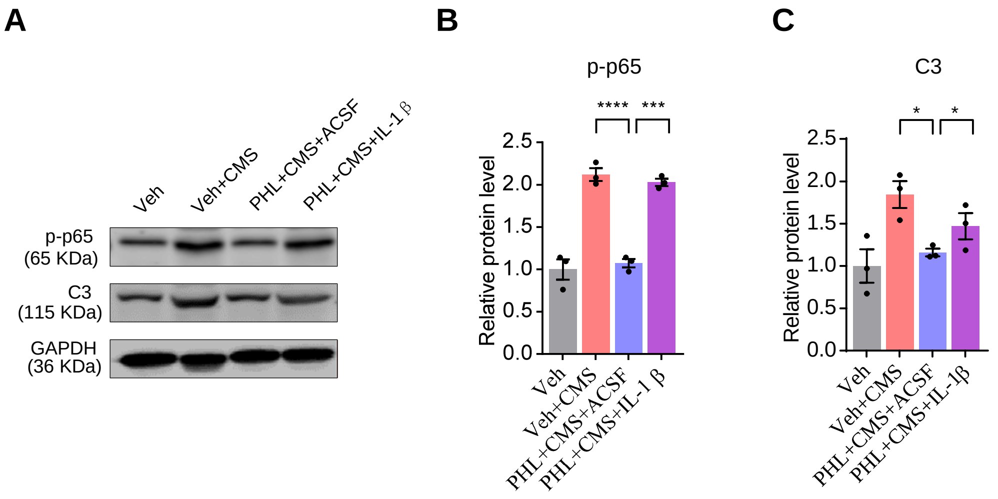

Figure S11. IL-1β increases the level of phosphorylation of p65 and C3 in the mPFC. (**A**) The level of p-p65 was detected by western blot in different groups. (**B-C**) Relative quantitative analysis of the level of p-p65 protein (**B**) and C3 protein (**C**) in different groups (n = 3 rats per group). Data are shown as mean ± SEM. One-way ANOVA with the Tukey's post hoc test (**B**, **C**). *$p < 0.05$, ***$p < 0.001$, ****$p < 0.0001$.

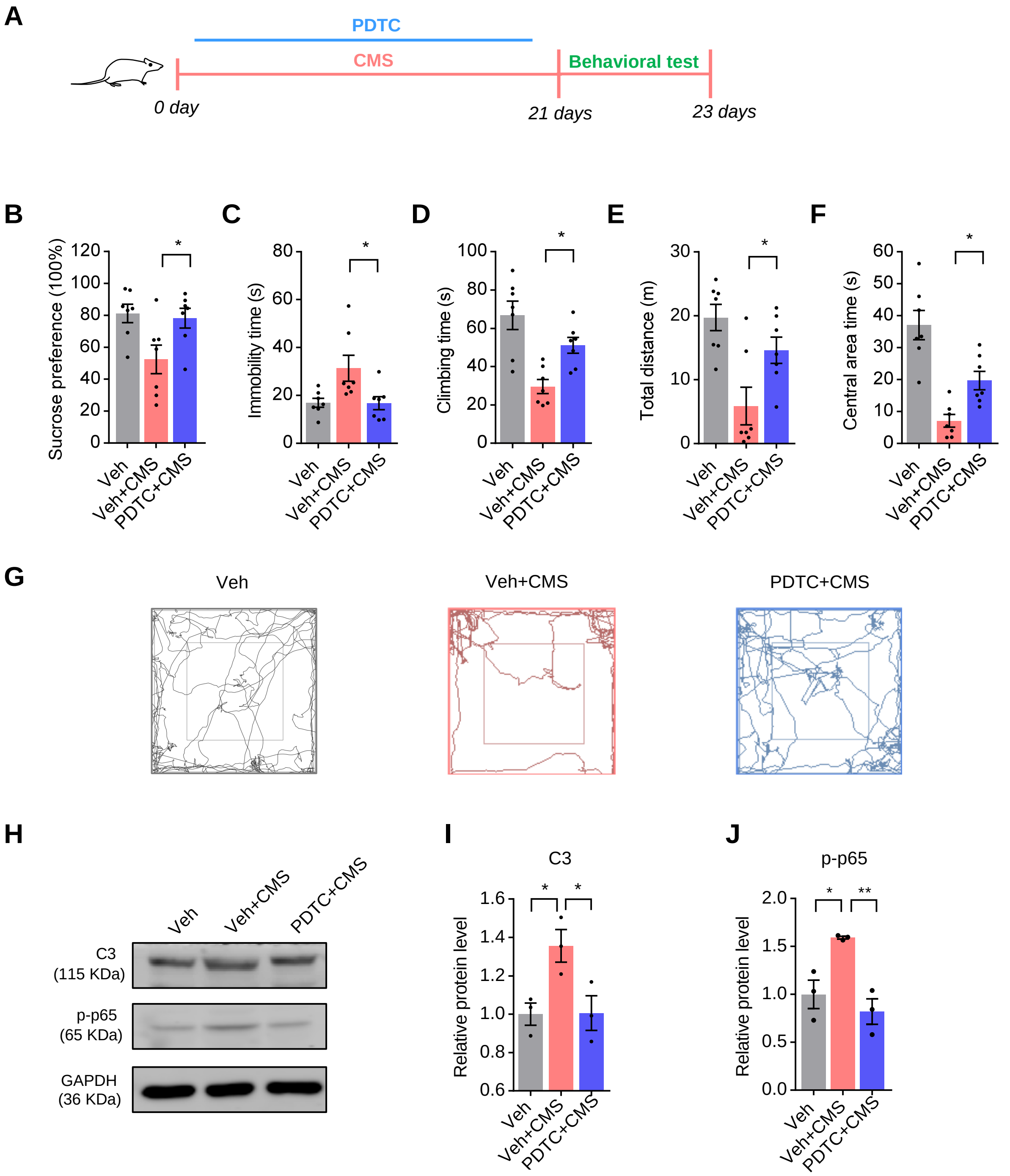

Figure S12. NF-kB pathway inhibitor PDTC improves the depression-like behaviors induced by CMS exposure. (**A**) Schematic procedure for investigating the PDTC on the depression-like effect in CMS rats. (**B**) Sucrose preference was measured in the CMS rats after the injection of PDTC (n = 7 rats per group). (**C-D**) The FST was evaluated in the CMS rats after the injection of PDTC (n = 7 rats per group). The immobility time spent in the FST (**C**). The climbing time spent in the FST (**D**). (**E-F**) The OFT was performed in the CMS rats after the injection of PDTC (n=7 rats per group). The total distance traveled in the open field area (**E**). Time spent in the center (**F**). (**G**) Representative animal traces of rat movement in the OFT. (**H**) The level of C3 and p-p65 protein were detected by western blot in different groups. (**I-J)** Relative quantitative analysis of the level of C3 (**I**) and p-p65 (**J**) protein level in different groups (n = 3 rats per group). Data are shown as mean ± SEM. One-way ANOVA with the Tukey's post hoc test (**B-F**, **I-J**). $^{*}p < 0.05$, $^{**}p < 0.01$.

Supplementary video captions

Video S1:

Super-resolution microscopy image and 3D-reconstruction showed the Iba-1$^+$ microglia (cyan) containing PSD95$^+$ puncta (green) and CD68 $^+$ (red) in the mPFC of Veh group rats. The same cell was shown in Figure 5H.

Video S2:

Super-resolution microscopy image and 3D-reconstruction showed the Iba-1$^+$ microglia (cyan) containing PSD95$^+$ puncta (green) and CD68 $^+$ (red) in the mPFC of Veh + CMS group rats. The same cell was shown in Figure 5H.

Video S3:

Super-resolution microscopy image and 3D-reconstruction showed the Iba-1$^+$ microglia (cyan) containing PSD95$^+$ puncta (green) and CD68 $^+$ (red) in the mPFC of Mino + CMS group rats. The same cell was shown in Figure 5H.

Video S4:

Super-resolution microscopy image and 3D-reconstruction showed the Iba-1$^+$ microglia (cyan) containing PSD95$^+$ puncta (green) and CD68 $^+$ (red) in the mPFC of Mino group rats. The same cell was shown in Figure 5H.

Video S5:

Super-resolution microscopy image and 3D-reconstruction showed the Iba-1$^+$ microglia (cyan) containing PSD95$^+$ puncta (green) and CD68 $^+$ (red) in the mPFC of Veh group rats. The same cell was shown in Figure 6F.

Video S6:

Super-resolution microscopy image and 3D-reconstruction showed the Iba-1$^+$ microglia (cyan) containing PSD95$^+$ puncta (green) and CD68 $^+$ (red) in the mPFC of Veh + CMS group rats. The same cell was shown in Figure 6F.

Video S7:

Super-resolution microscopy image and 3D-reconstruction showed the Iba-1$^+$ microglia (cyan) containing PSD95$^+$ puncta (green) and CD68 $^+$ (red) in the mPFC of PHL + CMS group rats. The same cell was shown in Figure 6F.

Video S8:

Super-resolution microscopy image and 3D-reconstruction showed the Iba-1$^+$ microglia (cyan) containing PSD95$^+$ puncta (green) and CD68 $^+$ (red) in the mPFC of PHL group rats. The same cell was shown in Figure 6F.

Supplementary References

1. Yang YT, Li YT, Liu B, Li CC, Liu ZJ, Deng JH, et al. Involvement of Scratch2 in GalR1-mediated depression-like behaviors in the rat ventral periaqueductal gray. Proc Natl Acad Sci U S A. 2021; 118: e1922586118.
2. Combs DJ, D'Alecy LG. Motor performance in rats exposed to severe forebrain ischemia: effect of fasting and 1, 3-butanediol. Stroke. 1987; 18: 503–11.
3. Chen J, Li Y, Wang L, Zhang Z, Lu D, Lu M, et al. Therapeutic benefit of intravenous administration of bone marrow stromal cells after cerebral ischemia in rats. Stroke. 2001; 32: 1005-11.
4. Jing B, Liu B, Li H, Lei J, Wang Z, Yang Y, et al. Within-subject test-retest reliability of the atlas-based cortical volume measurement in the rat brain: A voxel-based morphometry study. J Neurosci Methods. 2018; 307: 46-52.
5. Barrière DA, Magalhães R, Novais A, Marques P, Selingue E, Geffroy F, et al. The SIGMA rat brain templates and atlases for multimodal MRI data analysis and visualization. Nat Commun. 2019; 10: 5699.
6. Norris G, Derecki N, Kipnis J. Microglial Sholl analysis. Protocol Exchange. Doi: 10.1038/protex.2014.029.
7. Ferreira TA, Blackman AV, Oyrer J, Jayabal S, Chung AJ, Watt AJ, et al. Neuronal morphometry directly from bitmap images. Nat Methods 2014; 11: 982-4.
8. Hong S, Beja-Glasser VF, Nfonoyim BM, Frouin A, Li S, Ramakrishnan S, et al. Complement and microglia mediate early synapse loss in Alzheimer mouse models. Science. 2016; 352: 712-6.

9. Silva JM, McMahon M. The fastest Western in town: a contemporary twist on the classic Western blot analysis. J Vis Exp. 2014; 84: e51149.
